# Analytical modeling of novel equivalent circuits of double diode solar cell circuits using a special transcendental function approach

**DOI:** 10.1371/journal.pone.0313713

**Published:** 2024-11-14

**Authors:** Ziad M. Ali, Martin Ćalasan, Mostafa H. Mostafa, Shady H. E. Abdel Aleem

**Affiliations:** 1 Department of Electrical Engineering, College of Engineering in Wadi Alddawasir, Prince Sattam Bin Abdulaziz University, Wadi Alddawasir, Saudi Arabia; 2 Faculty of Electrical Engineering, University of Montenegro, Podgorica, Montenegro; 3 Department of Electrical Power and Machines, International Academy of Engineering and Media Science, Giza, Egypt; 4 Department of Electrical Engineering, Institute of Aviation Engineering and Technology, Giza, Egypt; SRM-RI: SRM Institute of Science and Technology (Deemed to be University) Research Kattankulathur, INDIA

## Abstract

Solar photovoltaic (PV) cell modeling is crucial to understanding and optimizing solar energy systems. While the single-diode model (PV_SDM_) is commonly used, the double-diode model (PV_DDM_) offers improved accuracy at a reasonable level of complexity. However, finding analytical closed-form solutions for the current-voltage (*I*-*U*) dependency in PV_DDM_ circuits has remained a challenge. This work proposes two novel configurations of PV_DDM_ equivalent circuits and derives their analytical closed-form solutions. The solutions are expressed in terms of the Lambert W function and solved using a special transcendental function approach called Special Trans Function Theory (STFT). The accuracy of the proposed equivalent circuits is demonstrated on two solar cells/modules, RTC-F and MSX-60, showing equal or better performance than the standard PV_DDM_ equivalent circuit. Further testing on a commercial solar panel under different irradiance and temperature conditions confirms the applicability of the proposed models. To address the parameter estimation problem, a novel metaheuristic algorithm, the chaotic honey-badger algorithm, is developed and evaluated. The results obtained validate the accuracy and practicality of the proposed PV_DDM_ equivalent circuit configurations.

## 1. Introduction

At present, the global situation is marked by a complex and pressing need for energy resources. The rapid development of industry, fossil fuel-based vehicles, and thermal power plants have emerged as major contributors to environmental pollution. Consequently, the key to addressing this challenge lies increasingly in the utilization of renewable energy sources (RESs) [1].

RESs have gained tremendous popularity in both the scientific community and in practical applications. These sources offer several important advantages, including availability in diverse environments, inexhaustible supply, and the distinct advantage of being environmentally friendly. From a technical standpoint, renewable energy can also help improve voltage conditions in the grid, reduce energy transmission losses, and enhance the overall security of electricity supply [2, 3]. Among REss, solar photovoltaic (PV) energy has emerged as one of the most prevalent sources. Several factors, such as the constant decline in the price of solar panels, the good predictability of solar power generation, and the inherent safety of solar technologies, drive the increasing adoption of solar energy. These factors are accompanied by many scientific publications exploring various aspects of solar panel performance, including parameter estimation, grid integration, production coordination, and integration with other energy generation facilities [4]. The standard equivalent circuit for modeling solar cells or panels typically consists of a single diode, a current source, and two resistors. The diode represents the *p*-*n* junction, the current source represents the photon-generated current, and the resistors account for various losses within the cell. This model is commonly referred to as the single-diode model (PV_SDM_). In addition to the SDM, the literature also describes more complex models, such as the double-diode model (PV_DDM_) [5] and the triple-diode model (PV_TDM_) [6], which incorporate two or three diodes in their respective equivalent circuits. In a mathematical sense, the PV_SDM_ is considered a 5th parameter problem [6, 7], while the PV_DDM_ and PV_TDM_ are 7th and 9th parameter problems, respectively [8]. This suggests that the PV_DDM_ has better accuracy than the PV_SDM_ but is less complex than the PV_TDM_. Besides, numerous works have focused on the estimation of the parameters for one-diode and two-diode models of solar cells [9–11]. However, an essential drawback of the standard PV_DDM_ equivalent circuit is that there is no analytical dependence on the output current and voltage [8]. This means an exact, closed-form expression relating the output current and voltage cannot be derived. Researchers have used approximate formulas to estimate the output current to address this issue. Some approaches include using an approximate formula based on the basic expression for the output current [12], employing an approximate formula expressed using two Lambert W functions, where some parameters are neglected [13], and employing an advanced iterative method using the Lambert W function, where some parameters are not neglected [8]. These approximate methods are necessary because the underlying mathematical dependence between the output current and voltage in the PV_DDM_ is not analytically tractable. These approaches reflect the complexity involved in accurately modeling the nonlinear behavior of solar cells using the PV_DDM_ equivalent circuit. The authors in this paper aim to develop modified PV_DDM_ equivalent circuits of solar cell circuits with 7 unknown parameters. Still, it will be possible to determine the analytical dependency between the output current and voltage. In this way, the solar cell’s modeling will be facilitated, the possibility of forming inverse mathematical dependencies will be provided, and the model’s accuracy regarding the application of approximate formulas for defining current-voltage (*I*-*U*) dependence will be improved.

A general feature of each solar cell model is that their parameters cannot be readily computed. Instead, a variety of approaches are employed for estimating the parameters of solar cells [10, 14–34]:

1. Analytical methods: These involve numerous approximations and simplifications [14, 27, 29].
2. Numerical methods: These are primarily based on the application of iterative techniques, which depend on the iteration steps as well as the initial conditions [33, 34].
3. Metaheuristic approaches: These have emerged as a vast research field in solar cell modeling [15–17, 21–24].

The cited references [10, 14–34] cover the breadth of these parameter estimation techniques. Metaheuristic algorithms can be further categorized based on the natural processes, mathematical principles, physical phenomena, or behavioral patterns that inspired their development.

**Fig 1:**
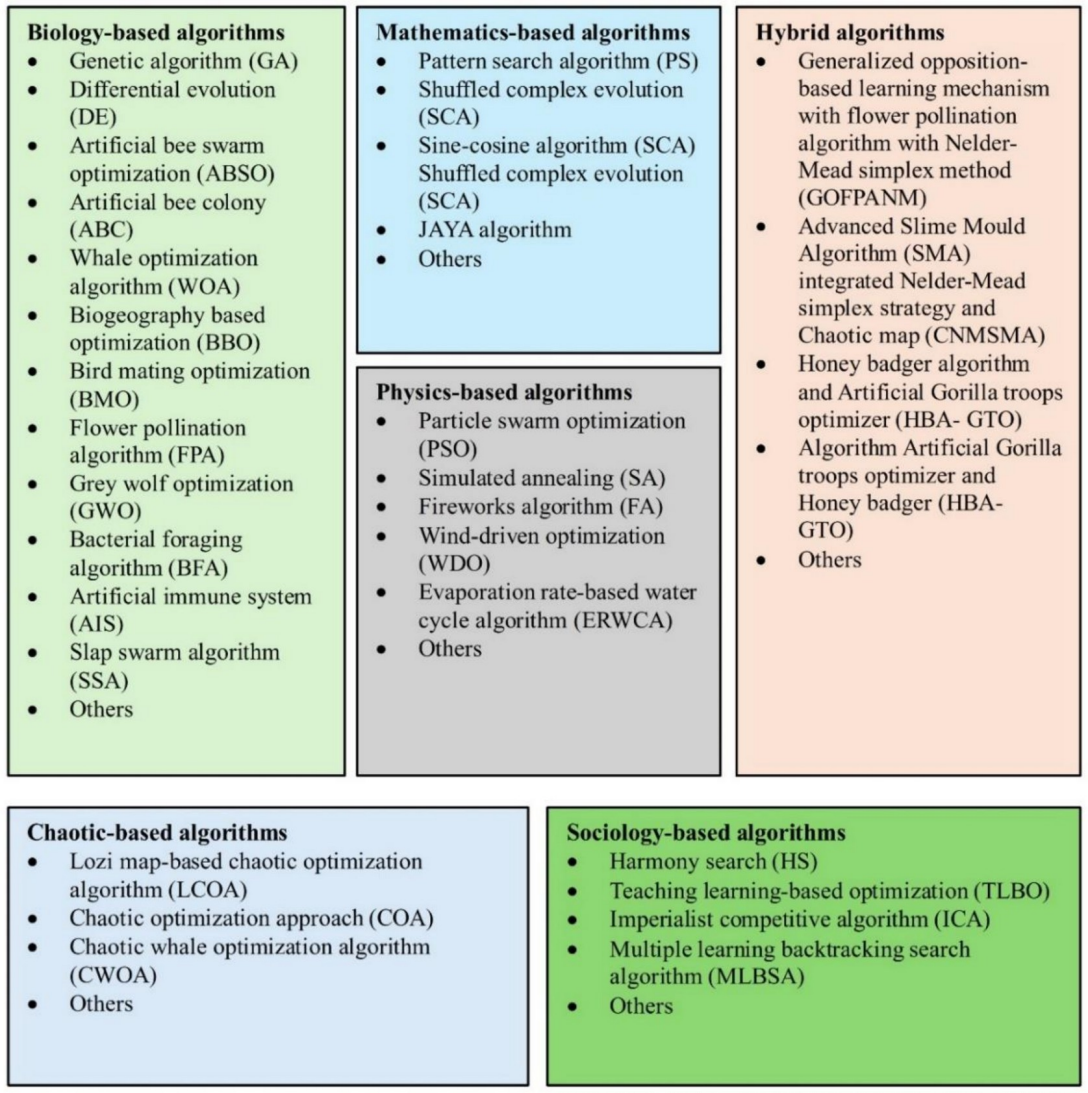
Overview of different metaheuristic algorithms used in PV parameter estimation and their corresponding full names and acronyms.

Fig 1 provides an overview of the different types of metaheuristic algorithms and their corresponding full names and acronyms. Generally speaking, the reliance on these diverse parameter estimation methods, from analytical to numerical to metaheuristic, underscores the inherent challenges in directly computing the parameters of solar cell models. The complexity of the underlying physics and nonlinear relationships involved necessitates using approximations, iterative techniques, and nature-inspired optimization algorithms to determine the model parameters effectively.

In this regard, one of this paper’s goals is to develop a novel metaheuristic algorithm for estimating the parameters of solar cell models. While numerous algorithms have been proposed in this field, an optimal solution has not yet been found, indicating that there is still room for scientific progress in this area. Observing the overall work, the main benefits and contributions of this paper can be summarized as follows:

1. Proposal of two new types of equivalent circuit schemes for PV_DDM_ of solar cells.
2. Derivation of analytical expressions in closed form for the *I*-*U* dependence. The solutions are expressed in terms of the Lambert W function and solved using a special transcendental function approach called Special Trans Function Theory (STFT).
3. Comparison of the output *I*-*U* curves with those obtained using standard double-diode solar cell models.
4. Application and validation of the proposed models on several test solar cells and panels and measured results.
5. Development of a new algorithm for estimating the parameters of solar diode cells and a comparative evaluation against other methods.

The work is organized as follows: The second section describes the standard equivalent circuit of the PV_DDM_ and reviews the parameter estimation methods available in the literature. The new PV_DDM_ circuits are proposed in the third section, and the corresponding *I*-*U* relations are derived. The fourth section introduces the newly developed metaheuristic algorithm for estimating solar cell parameters. The fifth section presents the parameter estimation results for two solar cells and one commercial solar panel. The experimental verification of the accuracy and efficiency of the proposed circuit and algorithm is discussed in the sixth section. A comparison of the proposed algorithm with other methods is presented in the seventh section. Finally, the conclusion highlights the main contributions of this work.

## 2. Standard solar cell double-diode-based equivalent circuit

**Fig 2:**
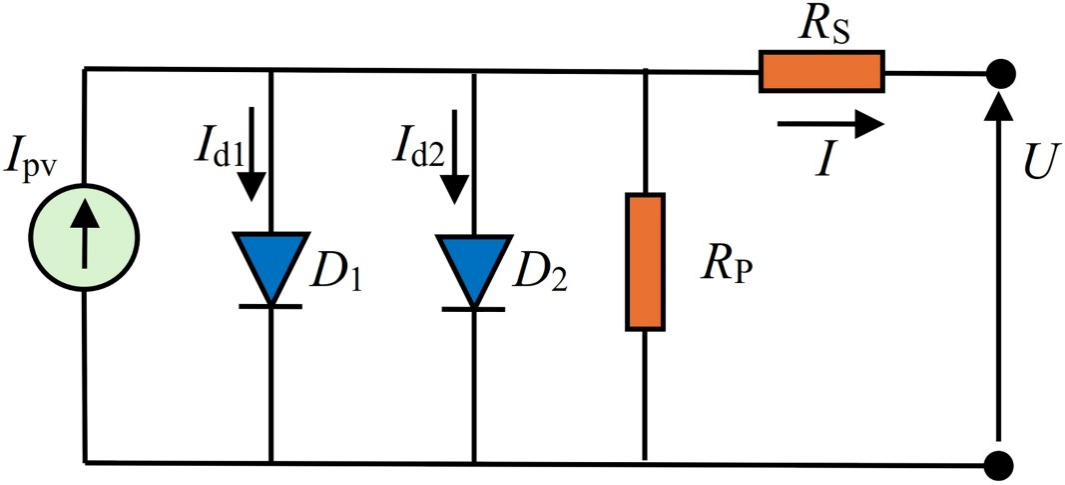
Standard PV_DDM_ equivalent circuit.

Fig 2 shows the standard diode circuit of solar cells. In this figure, *R*_*S*_ represents the series resistance, *R*_*P*_ represents the parallel resistance, and *D*_1_ and *D*_2_ are diodes, which describe the *pn* junction of solar cells. The current *I*_*pv*_ is photocurrent, i.e., the current generator. The *I*-*U* expression that governs this model is described as follows:

$$I=I_{pv}-I_{01}\left(e^{\frac{U+IR_{S}}{n_{1}\times V_{th}}}-1\right)-I_{02}\left(e^{\frac{U+IR_{S}}{n_{2}\times V_{th}}}-1\right)-\frac{U+IR_{S}}{R_{P}}$$ (1)

where *n*_1_ and *n*_2_ are the ideal factors of solar cells, and *I*_01_ and *I*_02_ are the inverse current diodes, while *V*_*th*_ is the thermal voltage. The relation between the output current and voltage of a solar cell is inherently nonlinear and transcendent. As noted in the literature [8], no analytical, closed-form expression has been developed to date that can directly relate the current and voltage in these models. The nonlinearity arises from the complex interplay of various physical phenomena within the solar cell, such as carrier generation, recombination, and transport processes. These underlying mechanisms give rise to the exponential and logarithmic relationships that govern the *I*-*U* characteristics of solar cells.

The root-mean-square error (*RMSE*) is used as an assessment criterion in estimating the parameters of solar cells. The mathematical expression for this criterion is expressed as follows and are presented in S1 File:

$$RMSE=\sqrt{\frac{1}{N_{meas}}\sum_{k=1}^{N_{meas}}{\left(I_{k}^{meas}-I_{k}^{sim}\right)}^{2}}$$ (2)

The question arises as to how the simulated current value (*I*_*sim*_) is calculated. In most research papers, this current is calculated as follows [12]:

$$I^{sim}=I_{pv}-I_{01}\left(e^{\frac{U_{meas}+I_{meas}R_{S}}{n_{1}\times V_{th}}}-1\right)-I_{02}\left(e^{\frac{U_{meas}+I_{meas}R_{S}}{n_{2}\times V_{th}}}-1\right)-\frac{U_{meas}+I_{meas}R_{S}}{R_{P}}$$ (3)

The previous formulation is not mathematically correct because the measured value of both voltage (*U*_*meas*_) and current (*I*_*meas*_) is used to calculate the simulated current value. Thus, according to the second approach proposed in [13], the simulated current value is calculated using the following expression:

$$\begin{array}{l} I^{sim}=\frac{R_{P}\left(I_{pv}+I_{01}+I_{02}\right)-U_{meas}}{R_{S}+R_{P}}-\frac{n_{1}}{2R_{S}}W\left(\frac{R_{S}R_{P}\left(I_{01}+I_{02}\right)}{n_{1}\left(R_{S}+R_{P}\right)}e^{\frac{R_{P}\left(R_{S}I_{pv}+R_{S}I_{01}+R_{S}I_{02}+U_{meas}\right)}{n_{1}\left(R_{S}+R_{P}\right)}}\right)\\ -\frac{n_{2}}{2R_{S}}W\left(\frac{R_{S}R_{P}\left(I_{01}+I_{02}\right)}{n_{2}\left(R_{S}+R_{P}\right)}e^{\frac{R_{P}\left(R_{S}I_{pv}+R_{S}I_{01}+R_{S}I_{02}+U_{meas}\right)}{n_{2}\left(R_{S}+R_{P}\right)}}\right) \end{array}$$ (4)

The simulated current value is calculated using two Lambert W functions. However, certain neglects are used to derive this expression mathematically [13], which means that the expression is not entirely mathematically correct.

The third approach for current calculation is based on employing the iterative method with the Lambert W function. Namely, by using the mentioned approach, the *I*-*U* for PV_DDM_ relation is presented in the following nonlinear form:

$$\alpha +\beta \cdot \text{exp}\left(\delta \cdot \Psi \right)=\Psi \cdot \text{exp}\left(\Psi \right)$$ (5)

where the current is expressed as follows:

$$I^{sim}=\frac{R_{P}}{R_{P}+R_{S}}\left(I_{pv}+I_{01}+I_{02}-\frac{U}{R_{P}}-\frac{n_{1}V_{th}}{R_{S}}\left(1+\frac{R_{S}}{R_{P}}\right)\cdot \Psi \right),$$ (6)

while the coefficients are as follows:

$$\alpha =\frac{R_{S}R_{P}I_{01}e^{\frac{U}{n_{1}V_{th}}}}{n_{1}V_{th}\left(R_{S}+R_{P}\right)}\left(e^{\frac{R_{S}R_{P}}{n_{1}V_{th}\left(R_{S}+R_{P}\right)}\left(I_{pv}+I_{01}+I_{02}-\frac{U}{R_{P}}\right)}\right),$$ (7)

$$\beta =\frac{R_{S}R_{P}I_{02}e^{\frac{U}{n_{2}V_{th}}}}{n_{1}V_{th}\left(R_{S}+R_{P}\right)}\left(e^{\frac{R_{S}R_{P}I_{02}}{n_{2}V_{th}\left(R_{S}+R_{P}\right)}\left(I_{pv}+I_{01}+I_{02}-\frac{U}{R_{P}}\right)}\right),$$ (8)

$$\delta =1-\frac{n_{1}}{n_{2}}$$ (9)

Eq (5) can be solved using the iterative procedure proposed in [8]. Therefore, based on this short review, it is clear that there is no accurate analytical solution for the voltage-current expression of the standard solar PV_DDM_.

## 3. Novel solar cell double-diode-based equivalent circuits

The efficiency of conventional solar cells remains relatively low, as only a small fraction of the incident solar energy is successfully converted into electricity. This is due to the numerous losses that occur within solar cell devices. These losses can be categorized into two main groups–intrinsic (or fundamental) and extrinsic losses [35]. The intrinsic losses include optical losses, thermalization losses, emission losses, and other fundamental phenomena. In contrast, the extrinsic losses encompass non-radiative recombination, series resistance, shunt resistance, and other system-level factors. In the standard double-diode model (PV_DDM_) equivalent circuit, these losses are typically represented by two resistance terms–one connected in parallel with the device terminals and another in series with the same. This section proposes two novel solar PV_DDM_ equivalent circuit topologies. Furthermore, in the third part of this section, analytical closed-form solutions are derived for the proposed models’ current voltage expressions. Developing these new equivalent circuit representations and their associated analytical *I*-*U* relationships aims to more accurately capture the intricate balance of solar cells’ intrinsic and extrinsic loss mechanisms. By refining the modeling approach, the ultimate goal is to enable more precise characterization and optimization of solar cell performance.

### 3.1 Proposed solar PV_DDM_ equivalent circuit 1 (DDMR_i_)

**Fig 3:**
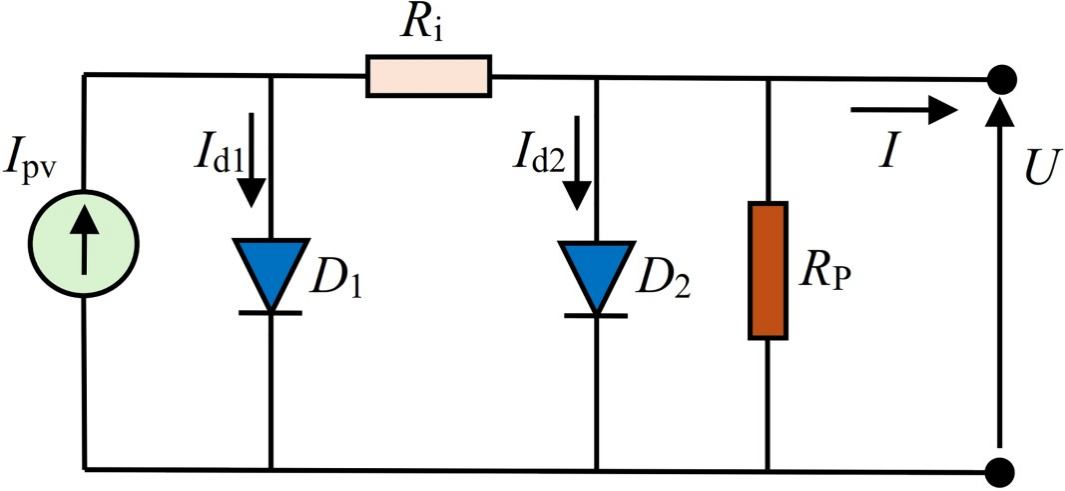
Proposed DDMR_i_ solar cell equivalent circuit.

The first solar cell PV_DDM_ equivalent circuit, abbreviated as DDMR_i_, is based on providing resistance in the branch between two diodes, as depicted in Fig 3. This circuit does not contain any series resistor like the standard PV_DDM_ circuit, but it implies the parallel one.

The *I*-*U* expression that governs this circuit is given as follows:

$$I_{pv}=I_{D1}+I_{D1}+\frac{U}{R_{P}}+I$$ (10)

where

$$\begin{array}{l} I_{D1}=I_{01}\left(e^{\frac{U_{D1}}{n_{1}V_{th}}}-1\right)=I_{01}\left(e^{\frac{U+R_{i}\left(I_{D2}+\frac{U}{R_{P}}+I\right)}{n_{1}V_{th}}}-1\right)\\ I_{D2}=I_{02}\left(e^{\frac{U}{n_{2}V_{th}}}-1\right) \end{array}$$ (11)

After certain mathematical operations, these equations are reduced to the form:

$$I=I_{pv}+I_{01}-I_{02}\left(e^{\frac{U}{n_{2}V_{th}}}-1\right)-\frac{U}{R_{P}}-\frac{n_{1}V_{th}}{R_{i}}\left(ϒ\right)$$ (12)

where ϒ is the solution of the Lambert W function expressed as follows:

ϒ=α⋅exp(−ϒ) (13)

so that

$$\alpha =\frac{R_{i}I_{01}}{n_{1}V_{th}}\cdot \text{exp}\left(\frac{U+R_{i}\left(I_{pv}+I_{01}\right)}{n_{1}V_{th}}\right)$$ (14)

### 3.2 Proposed solar PV_DDM_ equivalent circuit 2 (DDMR_D_)

**Fig 4:**
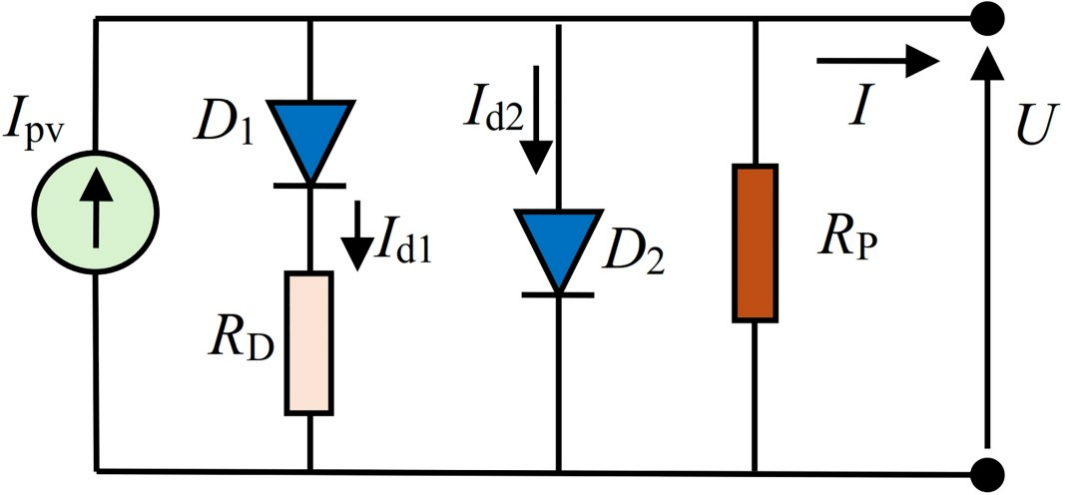
Proposed DDMR_D_ solar cell equivalent circuit (variant#1).

The second solar PV_DDM_ equivalent circuit, abbreviated as DDMR_D_, is based on providing a series resistance with *D*_1_, variant#1. Also, like the DDMR_i_, this circuit does not contain a series resistor like the standard PV_DDM_ circuit but includes a parallel resistance. The proposed equivalent circuit is depicted in Fig 4.

The *I*-*U* characteristic of this circuit is given as:

$$I_{pv}=I_{01}\left(e^{\frac{U-R_{D}\left(I_{pv}-I_{D2}-\frac{U}{R_{P}}-I\right)}{n_{1}V_{th}}}-1\right)+I_{02}\left(e^{\frac{U}{n_{2}V_{th}}}-1\right)+\frac{U}{R_{P}}+I$$ (15)

After certain mathematical operations, these equations are reduced to the form:

$$I=I_{pv}+I_{01}-I_{02}\left(e^{\frac{U}{n_{2}V_{th}}}-1\right)-\frac{U}{R_{P}}-\frac{n_{1}V_{th}}{R_{D}}\left(ϒ\right)$$ (16)

where ϒ is the solution of the Lambert W function expressed as follows:

ϒ=α⋅exp(−ϒ) (17)

so that

$$\alpha =\frac{R_{D}I_{01}}{n_{1}V_{th}}\cdot \text{exp}\left(\frac{U+R_{D}I_{01}}{n_{1}V_{th}}\right)$$ (18)

**Fig 5:**
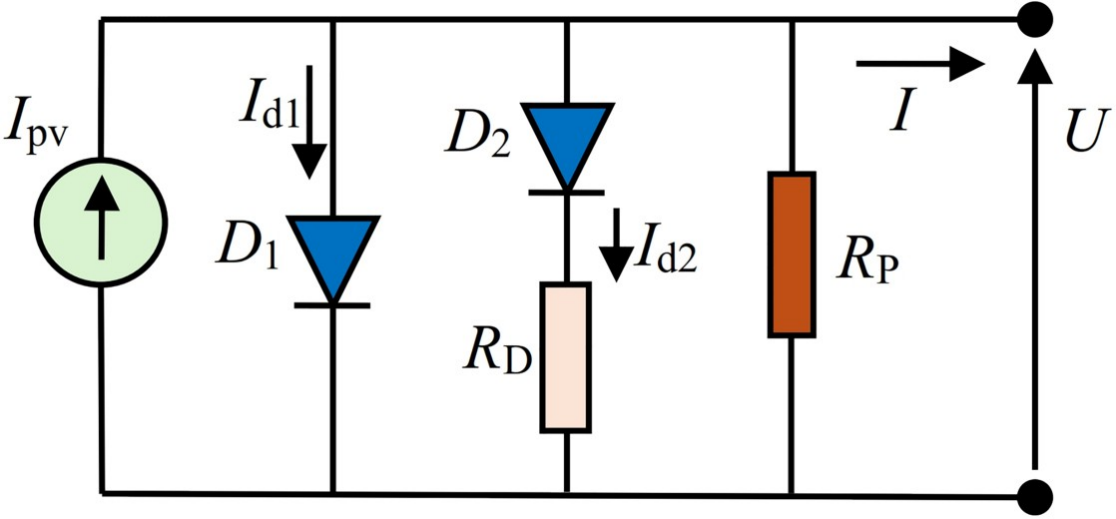
Proposed DDMR_D_ solar cell equivalent circuit (variant#2).

The proposed equivalent circuit DDMR_D_ shown in Fig 4 can also be presented by providing a series resistance with *D*_2_, variant#2, as depicted in Fig 5.

In this case, the expression for current has the following form:

$$I=I_{pv}+I_{02}-I_{01}\left(e^{\frac{U}{n_{1}V_{th}}}-1\right)-\frac{U}{R_{P}}-\frac{n_{2}V_{th}}{R_{D}}\left(ϒ\right)$$ (19)

where ϒ is the solution of the Lambert W function expressed as follows:

ϒ=α⋅exp(−ϒ) (20)

so that

$$\alpha =\frac{R_{D}I_{02}}{n_{2}V_{th}}\cdot \text{exp}\left(\frac{U+R_{D}I_{02}}{n_{2}V_{th}}\right)$$ (21)

### 3.3 Analytical expressions for currents as function in voltages for both proposed DDM_R_ and DDM_D_

For both proposed equivalent circuits, the *I*-*U* relationship is represented via the Lambert W function. The Lambert W function can be solved using iterative methods (such as Halley and Fritsch iterations), many software packages that implement this function (MATLAB, Maple, and so on), or analytically. Currently, three analytical approaches to the solution can be found in the literature. First, if the value of the function argument is large (in our case, denoted by α) [36], the analytical expression for the variable ϒ will have the form:

$$ϒ=L_{1}-L_{2}+\overset{\infty }{\underset{l=0}{\sum }}\overset{\infty }{\underset{m=1}{\sum }}\frac{{\left(-1\right)}^{l}\left[\begin{array}{c} l+m\\ l+1 \end{array}\right]}{m!}L_{1}^{-l-m}L_{2}^{m}$$ (22)

where *L*_1_ = ln(*α*), *L*_2_ = ln(ln(*α*)), $\left[\begin{array}{c} l+m\\ l+1 \end{array}\right]$ are non-negative Stirling numbers of the first kind.

Second, the Lambert W function can be solved by applying the Taylor series [36, 37] as follows:

$$ϒ=\overset{M}{\underset{n=1}{\sum }}\frac{{\left(-n\right)}^{n-1}}{n!}\alpha^{n},$$ (23)

where M is a positive integer.

Third, the Lambert W function can be solved by applying the STFT [36–39] as follows:

$$ϒ=\alpha \frac{\sum_{n=0}^{M}\frac{\alpha^{n}{\left(M-n\right)}^{n}}{n!}}{\sum_{n=0}^{M+1}\frac{\alpha^{n}{\left(M+1-n\right)}^{n}}{n!}}$$ (24)

According to the comparative analysis presented in [37], the STFT approach to solving the Lambert W function in the proposed double-diode solar cell equivalent circuits demonstrates an unparalleled advantage in terms of accuracy and applicability across various values of *α* compared to the other two analytical methods. The key findings from the comparative analysis are:

1. Accuracy: The STFT-based solution exhibits superior accuracy in modeling the *I*-*U* relationship of the double-diode solar cell equivalent circuits, outperforming the direct analytical solution and the series expansion method.
2. Applicability: The STFT approach is highly versatile and can accurately capture the *I*-*U* characteristics across a broader range of parameter values. The other analytical methods demonstrated limited applicability and accuracy in specific parameter regimes.
3. Computational efficiency: Unlike the other analytical approaches, the STFT-based solution provides a closed-form expression that can be evaluated efficiently without the need for computationally intensive iterative numerical solvers.

Therefore, the closed-loop analytical expressions for both proposed circuits have the following forms:

- For DDMR_i_ shown in Fig 3
$$\begin{array}{l} I=I_{pv}+I_{01}-I_{02}\left(e^{\frac{U}{n_{2}V_{th}}}-1\right)-\frac{U}{R_{P}}\\ -I_{01}e^{\frac{U+R_{i}\left(I_{pv}+I_{01}\right)}{n_{1}V_{th}}}\frac{\sum_{n=0}^{M}\frac{{\left(\frac{R_{i}I_{01}}{n_{1}V_{th}}\cdot e^{\frac{U+R_{i}\left(I_{pv}+I_{01}\right)}{n_{1}V_{th}}}\right)}^{n}{\left(M-n\right)}^{n}}{n!}}{\sum_{n=0}^{M+1}\frac{{\left(\frac{R_{i}I_{01}}{n_{1}V_{th}}\cdot e^{\frac{U+R_{i}\left(I_{pv}+I_{01}\right)}{n_{1}V_{th}}}\right)}^{n}{\left(M+1-n\right)}^{n}}{n!}} \end{array}$$ (25)
- For DDMR_D_ shown in Fig 4
$$I=I_{pv}+I_{01}-I_{02}\left(e^{\frac{U}{n_{2}V_{th}}}-1\right)-\frac{U}{R_{P}}-I_{01}\cdot e^{\frac{U+R_{D}I_{01}}{n_{1}V_{th}}}\frac{\sum_{n=0}^{M}\frac{{\left(\frac{R_{D}I_{01}}{n_{1}V_{th}}\cdot e^{\frac{U+R_{D}I_{01}}{n_{1}V_{th}}}\right)}^{n}{\left(M-n\right)}^{n}}{n!}}{\sum_{n=0}^{M+1}\frac{{\left(\frac{R_{D}I_{01}}{n_{1}V_{th}}\cdot e^{\frac{U+R_{D}I_{01}}{n_{1}V_{th}}}\right)}^{n}{\left(M+1-n\right)}^{n}}{n!}}$$ (26)
- Finally, for DDMR_D_ shown in Fig 5
$$I=I_{pv}+I_{02}-I_{01}\left(e^{\frac{U}{n_{1}V_{th}}}-1\right)-\frac{U}{R_{P}}-I_{02}e^{\frac{U+R_{D}I_{02}}{n_{2}V_{th}}}\frac{\sum_{n=0}^{M}\frac{{\left(\frac{R_{D}I_{02}}{n_{2}V_{th}}e^{\frac{U+R_{D}I_{02}}{n_{2}V_{th}}}\right)}^{n}{\left(M-n\right)}^{n}}{n!}}{\sum_{n=0}^{M+1}\frac{{\left(\frac{R_{D}I_{02}}{n_{2}V_{th}}e^{\frac{U+R_{D}I_{02}}{n_{2}V_{th}}}\right)}^{n}{\left(M+1-n\right)}^{n}}{n!}}$$ (27)

## 4. Chaotic honey badger algorithm (CHBA)

The inspiration for the biomimetic Honey Badger Algorithm (HBA) is derived from honey badgers’ foraging behavior in their quest to locate food sources. In this algorithm, the population is represented by a collection of honey badgers (HBs), each characterized by its spatial position denoted with *x*_*i*_: *x*_*i*_ = [*x*_*i*_^1^, *x*_*i*_^2^,…, *x*_*i*_^*D*^], where *D* represents the number of optimization variables, and *i* = 1, 2, 3,…, *N*, where *N* stands for the number of HBs in the population. The HB is known for its tenacious and resourceful nature when seeking sustenance. This adaptive behavior serves as the guiding principle for the HBA, where the algorithm emulates the HBs’ strategies to efficiently explore the problem space and identify optimal solutions. The algorithm taps into the HBs’ instinctive abilities to navigate complex environments, overcome obstacles, and locate valuable resources. The HBA aims to harness HBs’ problem-solving prowess through this biomimetic approach and translate it into an effective metaheuristic optimization technique. The algorithm’s design is inspired by the HBs’ remarkable persistence, adaptability, and resourcefulness in their pursuit of food, which are valuable attributes for solving complex optimization problems [40].

Before starting the iteration process, the population is randomly initialized in the original HBA algorithm, considering that each HB’s position is between lower and upper bound (*L*_*B*_) and (*U*_*B*_). The initialization is carried out using the following equation:

$$x_{i}=rand\left(U_{B}-L_{B}\right)+L_{B}$$ (28)

where *rand* is a vector of random numbers between 0 and 1.

This paper proposes the modified version of the chaotic-HBA, in which the population is not initialized randomly according to the previous equation. Namely, the random initialization does not provide the optimal initial population of the algorithm and may lead to slower convergence, i.e., the optimal solution will be reached in a higher number of iterations [41]. The paper’s main idea is to apply a chaotic algorithm based on Gauss-iterated maps to generate the initial positions of HBs. This approach ensures that the initial solution is not generated randomly but is a product of specific optimization processes and is supposed to lead to the faster convergence of the Chaotic-HBA (CHBA). The initialization of the population, according to the Gauss mapping chaotic approach, is carried out as follows:

$$\begin{array}{l} y_{1}=rand,\\ y_{i+1}=\text{exp}\left(-\alpha {y_{i}}^{2}\right)+\beta ,\\ x_{i}=y_{i}\cdot \left(U_{B}-L_{B}\right)+L_{B},\;i=1,2,\ldots ,m, \end{array}$$ (29)

where *rand* represents a vector of random numbers in the interval [0, 1]. *α* and *β* take values of 4.9 and -0.58, respectively.

After the initialization process, the digging and honey phases are iteratively repeated until the maximum number of iterations is reached. In both phases, the position of each HB is updated according to corresponding mathematical equations. Firstly, the digging phase comes, and the positions are updated as follows:

$$x_{new}=x_{prey}+\left(F\cdot \beta \cdot I\cdot x_{prey}\right)+\left(F\cdot r_{3}\cdot \alpha \cdot d_{i}\cdot \left|\text{cos}\left(2\pi r_{4}\right)\cdot \left[1-\text{cos}\left(2\pi r_{5}\right)\right]\right|\right).$$ (30)

The terms in (30) have the following meanings: *x*_*new*_ denotes the updated position of the HB, *x*_*prey*_ is the best HB so far, *β* is the ability of the HB to get food (set to 6), *r*_2_, *r*_3_, *r*_4_, and *r*_5_ and *r*_6_ are random numbers between 0 and 1. Also, the intensity of the prey is denoted with *I*_*i*_ and is expressed in (31), source strength is *S* and is expressed in (32), the distance between prey and the *i*th badger is *d*_*i*_ and is described in (33), *α* is the density factor (*t* represents the current iteration and *t*_*max*_ for the maximum number of iterations) and is described in (34), and *F* is a flag that is expressed in (35).


$$I_{i}=r_{2}\left(\frac{S}{4\pi {d_{i}}^{2}}\right),$$ (31)

$$S={\left[x_{i}-x_{i+1}\right]}^{2},$$ (32)

$$d_{i}=x_{prey}-x_{i},$$ (33)

$$\alpha =e^{-\frac{t}{t_{\text{max}}}},$$ (34)

$$F=\left\{\begin{array}{ll} 1, & \text{if}\;r_{6}\leq 0.5\\ -1, & \text{else} \end{array}\right.,$$ (35)

Secondly, the digging phase is followed by the honey phase, which is presented with the following simple equation:

$$x_{new}=x_{prey}+F\cdot r_{7}\cdot \alpha \cdot d_{i},$$ (36)

where *r*_7_ denotes a random number between 0 and 1.

Once the CHBA’s iterative optimization process has been completed, the position of the HB with the lowest fitness function value is identified as the global optimal solution.

**Fig 6:**
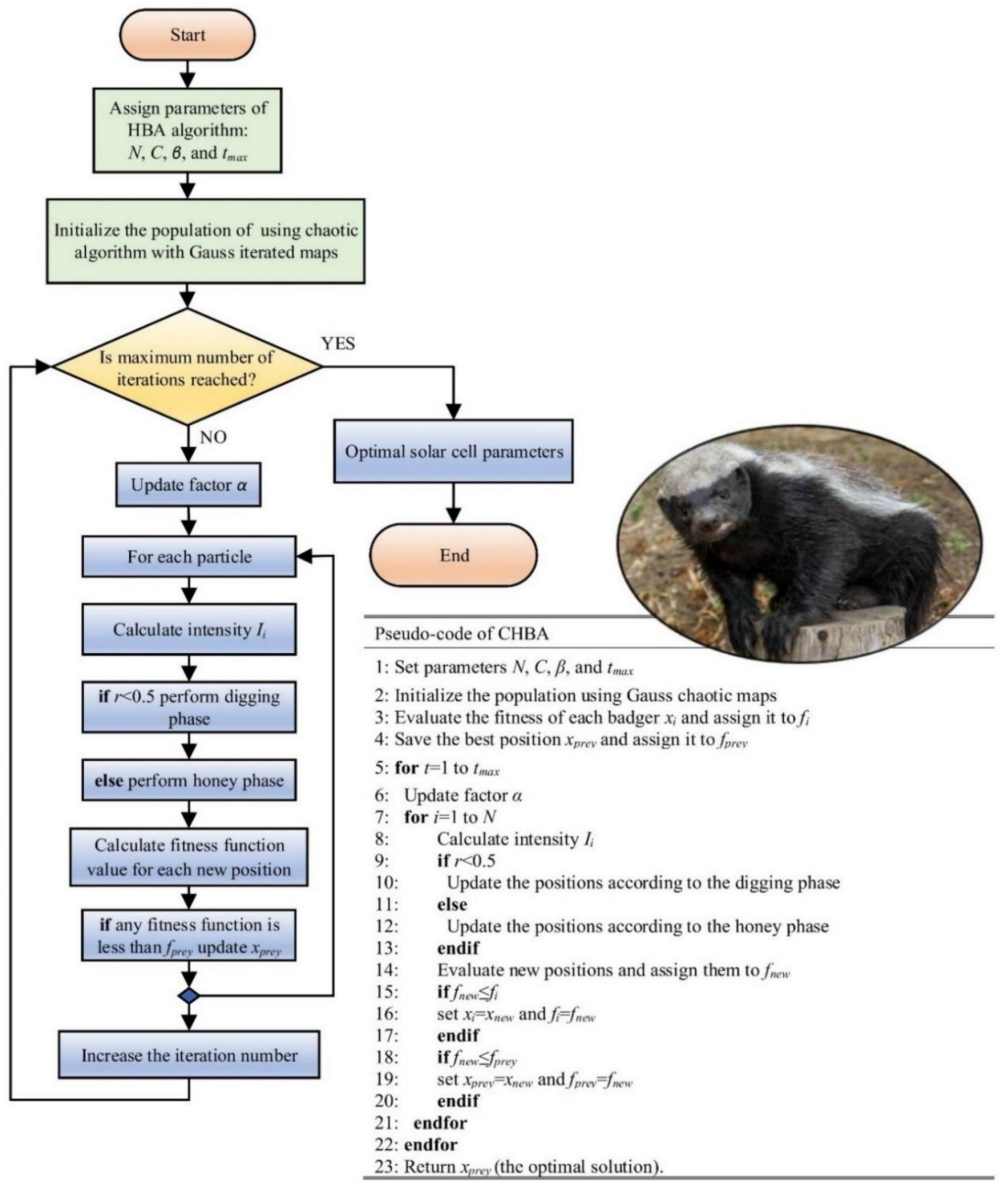
Flowchart and pseudocode of the CHBA.

The pseudocode outlines the key steps of the CHBA, and its flowchart is given in Fig 6 to provide a clear understanding of the steps involved in the proposed algorithm.

## 5. Numerical results

The findings of the parameter estimation for the standard and proposed equivalent circuits of the PV_DDM_ of solar cells are presented in this section. The estimation process involves an *RMSE* minimization problem, as shown in (2). All calculations were performed using MATLAB, version R2019b. The analysis focused on the RTC-F solar cell and two solar modules, SOLAREX MSX–60 PV and Photowatt-PWP201, to evaluate the accuracy of the proposed equivalent circuit and algorithm. According to the relevant solar cell parameter estimation literature, the estimated parameter values for the utilized solar cells/modules are presented in Tables 1 to 3.

**Table 1:** Numerical *RMSE* results in parameters estimation of RTC-F solar cell.

Method	Ref./First author	Full title of the algorithm	Short title	*I*_*pv*_ (A)	*I*_01_ (μA)	*n*_1_	*R*_*S*_ (Ω)	*R*_*P*_ (Ω)	*I*_02_ (μA)	*n*_2_	*RMSE*
1	[16]–W. Zhou	Random learning gradient-based optimizer	RLGBO	0.760778	0.591623	2.00000	0.0366529	54.9032	0.244765	1.457700	0.000761275197072
2		Gradient based optimizer	GBO	0.76078	0.24333	1.45833	0.0366117	54.6022	0.342131	1.863020	0.000764790289337
3		Forensic-based investigation algorithm	FBI	0.76067	0.103207	1.94432	0.0357744	58.4304	0.355873	1.492020	0.000832728243615
4		Spherical evolution algorithm	SE	0.760777	0.559451	1.79902	0.0352389	66.6324	0.263122	1.474360	0.001016788363008
5		Neural network algorithm	NNA	0.408016	0.532819	1.93017	0.0433618	56.2381	0.429974	1.567680	0.296459602187291
6		Backtracking search algorithm	BSA	0.761121	0.406717	1.50491	0.0354838	53.3551	0	1.636770	0.000981876634751
7		Generalized oppositional teaching learning-based optimization	GOTLBO	0.760678	0.270925	1.47106	0.0359831	59.01	0.317109	1.800420	0.000824909343625
8		Improved JAYA	IJAYA	0.760678	0.305984	1.47576	0.0366212	51.2958	0	1.606520	0.000806662934442
9	[17]–W. Long	Enhanced adaptive butterfly optimization algorithm	EABOA	0.76082865	0.25072	1.45988481	0.0366266	55.3660129	0.72069	1.99997318	0.002525316075064
10		Chaotic whale optimization algorithm	CWOA	0.76077	0.2415	1.45651	0.03666	55.2016	0.6	1.9899	0.000842359601137
11		Multiple learning backtracking search algorithm	MLBSA	0.7608	0.22728	1.4515	0.0367	55.4612	0.73835	2	0.000759993766175
12		Comprehensive learning particle swarm optimizer	CLPSO	0.7607	0.25843	1.4625	0.0367	57.9422	0.38615	1.9435	0.000788578623039
13		Harmony search	HS	0.76176	0.12545	1.49439	0.03545	46.82696	0.2547	1.49989	0.001053810153379
14		Biogeography-based optimization	BBO-M	0.76083	0.59115	1.45798	0.03664	55.0494	0.24523	2	0.210567000608764
15		Improved whale optimization algorithm	IWOA	0.7608	0.1667	1.6086	0.0361	55.2366	0.2323	1.4658	0.000795893464253
16		Pattern search	PS	0.7602	0.9889	1.6	0.032	81.3008	0.0001	1.192	0.010026894378555
17		Simulated annealing	SA	0.7623	0.4767	1.5172	0.0345	43.1034	0.01	2	0.010231285512016
18		Artificial bee swarm optimization algorithm	ABSO	0.76078	0.26713	1.46512	0.03657	54.6219	0.38191	1.98344	0.000767619556505
19		Improved JAYA algorithm	IJAYA	0.7601	0.005045	1.2186	0.0376	77.8519	0.75094	1.6247	0.000985664694752
20		Grey wolf optimizer	GWO	0.7620374	0.840453	1.58866804	0.031082963	63.0140077	0.33844	1.92985773	0.002278296398832
21		Modified grey wolf optimizer	mGWO	0.761236715	0.2503	1.48774762	0.035338346	49.413513	0.33551	1.93500106	0.056877728955935
22		Opposition-based sine cosine algorithm	OBSCA	0.79438075	0.528673	1.52753098	0.034681742	23.9696779	0.0444	2	0.022638796746720
23		Whale optimization algorithm	WOA	0.761034616	0	1.08904987	0.037253011	47.7236005	0.26394	1.46113223	0.000824220935469
24		Harris Hawks optimization	HHO	0.761304829	0.92747	1.67597805	0.026831714	92.6234167	0.92275	1.6869653	0.003445252502300
25		Inspired grey wolf optimizer	IGWO	0.761054815	0.14612	1.42331691	0.036494955	53.6695771	0.65612	1.77532836	0.000817424543035
26		Butterfly optimization algorithm	BOA	0.780771915	0.89987	1.47604191	0.03875443	34.5559104	0.29385	1.99100232	0.272466536462798
27	[18]–X. Weng	Generalized oppositional teaching learning-based optimization	GOTLBO	0.760705	0.28061	1.470062	0.036469	54.81858	0.170945	1.844222	0.000772038355673
28		Multiple learning backtracking search algorithm	MLBSA	0.760782	0.25558	1.461423	0.03661	54.78642	0.467554	1.981769	0.000763414773681
29		Improved JAYA	IJAYA	0.760678	0.62000	1.805871	0.036188	56.30568	0.340398	1.486475	0.030056220267356
30		Algorithm based on FPA, Nelder-Mead simplex, and the GOBL mechanism	GOFPANM	0.760781	0.74934	2	0.03674	55.48541	0.225975	1.451017	0.000757587784357
31		Orthogonally adapted Harris Hawks optimization	EHHO	0.759889	0.11494	1.482887	0.036409	73.47372	0.222945	1.486806	0.001050190600436
32		Dynamic sine-cosine spherical evolution algorithm	DSCSE	0.76078	0.24480	1.457724	0.036656	55.02486	0.588555	1.998663	0.000761115179919
33		Spherical evolution	SE	0.759561	0.12620	1.396962	0.039314	63.48457	0.426676	1.884294	0.001463976225018
34		Laplace’s cross search mechanism (LCS) and the Nelder-Mead simplex method (NMs)	LCNMSE	0.760781	0.74933	2	0.03674	55.48542	0.22598	1.451017	0.000757590481049
35	[19]–Y. Fan	Delayed dynamic step mechanism based on SFLA algorithm	DDSFLA	0.7608	0.2931	1.4730	0.0365	54.3710	0.2271	2.0000	0.000772702898061
36		Generalized oppositional teaching learning-based optimization	GOTLBO	0.7607	0.2427	1.6685	0.0365	54.9130	0.1972	1.4479	0.000777753987950
37		Improved JAYA algorithm	IJAYA	0.7607	0.2656	1.4664	0.0366	52.7410	0.0955	1.6935	0.000785997831117
38		Particle swarm optimization	PSO	0.7606	0.3495	1.4897	0.0361	61.3690	0.0950	2.0000	0.000851443503973
39		Salp swarm algorithm	SSA	0.7603	0.2341	1.6764	0.0370	59.8600	0.1848	1.4400	0.000847937703524
40		Grey wolf optimizer	GWO	0.7612	0.0926	1.3912	0.0367	53.7900	0.8229	1.7544	0.000853362405244
41		Cuckoo search	CS	0.7610	0.8913	1.9266	0.0369	55.8260	0.1952	1.4411	0.000962816251613
42		Whale optimization algorithm	WOA	0.7606	0.0106	1.9999	0.0345	63.9400	0.4795	1.5222	0.001047472286207
43		Shuffled frog-leaping algorithm	SFLA	0.7632	0.4549	1.5373	0.0338	46.3390	0.2159	1.6308	0.002317219495897
44		Dragon fly algorithm	DA	0.7618	0.0753	1.3684	0.0374	63.8180	0.4456	1.6901	0.003062682411637
45		Sine cosine algorithm	SCA	0.7797	0.0932	1.6436	0.0355	78.0850	0.8180	1.5857	0.014155828517942
46	[20]–S. Yang	Micro-charge field effect P systems optimization algorithm	MFE-POA	0.76078	0.22604	1.45111	0.03673	55.5295	0.73871	1.99508	0.000758034652869
47		NO MCI rule in P systems optimization algorithm	NoM-POA	0.76077	0.61611	1.90985	0.03668	55.8878	0.21453	1.44797	0.000761974721266
**48**		**Bird mating optimizer**	**BMO**	**0.76078**	**0.21110**	**1.44533**	**0.03682**	**55.8081**	**0.87688**	**1.99997**	**0.000754742709027**
49		Artificial bee swarm optimization	ABSO	0.76078	0.26713	1.46512	0.03657	54.6219	0.38191	1.98152	0.000765494526404
50		Teaching–learning-based artificial bee colony	TLABC	0.76081	0.42394	1.9075	0.03667	54.66797	0.24011	1.45671	0.000762846636345
51		Innovative global harmony search	IGHS	0.7608	0.9731	1.9213	0.0369	53.837	0.1679	1.4281	0.000837657942016
52		Grouping-based global harmony search	GGHS	0.76056	0.37014	1.49638	0.03562	62.7899	0.13504	1.92998	0.000877968042092
53		Harmony search	HS	0.76176	0.12545	1.49439	0.03545	46.8270	0.25470	1.49989	0.001053809595341
54		Pattern search	PS	0.7602	0.9889	1.6000	0.0320	81.301	0.0001	1.1920	0.010026886757100
55		Simulated annealing	SA	0.7623	0.4767	1.5172	0.0345	43.103	0.0100	2.0000	0.010231336195346
56	[21]–Y. Liu	Shuffled frog leaping algorithm	SFLA	0.76078	0.322175	1.757630	0.036628	54.7012	0.209785	1.448838	0.000765462297184
57		Artificial bee colony	ABC	0.75157	0.600730	1.604813	0.038274	71.3983	0.099330	1.446810	0.016264040446829
58		Particle swarm optimization	PSO	0.76138	1.0	1.616039	0.031671	31.4733	0.631352	1.968863	0.008542856194863
59		Multiple learning backtracking search algorithm	MLBSA	0.76078	0.225987	1.451022	0.036740	55.4853	0.749236	1.999999	0.000757594479417
60		Improved JAYA	IJAYA	0.76075	0.113922	1.395920	0.038029	50.2874	0.547259	1.788095	0.000841572789829
61		Generalized oppositional teaching learning-based optimization	GOTLBO	0.76076	0.271619	1.466542	0.036538	54.8475	0.382672	1.996602	0.000766511369047
62		Chaotic whale optimization algorithm	CWOA	0.76235	0.480764	1.657157	0.035767	62.5837	0.143380	1.437866	0.001564462109146
63		Enhanced Harris Hawks optimization	EHHO	0.76081	0.266555	1.873319	0.036603	53.3652	0.257960	1.462644	0.000766512254895
64		Shuffled frog leaping with memory poo	SFLBS	0.76077	0.775995	2.0	0.036755	55.5496	0.228580	1.449857	0.005244313465689
65	[10]–F. E. Ndi	Equilibrium optimizer	EO	0.76792	0.39999	2	0.03659	54.17614	0.26605	1.46451	0.006348583736994
66		Enhanced vibrating particle system algorithm	EVPS	0.7607	0.29749	1.4749	0.0363	55.8827	0.2504	1.9726	0.000778574890709
67		Innovative global harmony search	IGHS	0.76079	0.9731	1.92126	0.0369	56.8368	0.16791	1.42814	0.000756490371760
58		Combinatorial particle swarm optimization	CPSO	0.762321	0.297108	1.476035	0.035601	45.547533	0.710454	1.998103	0.004340101762747
69		Artificial bee colony	ABC	0.7609	2.69	1.467	0.0369	56.8368	2.8198	1.8722	0.712845677562795
70		Artificial bee colony optimization	ABCO	0.7608	0.0407	1.4495	0.0364	53.7804	0.2874	14.885	0.256799429697175
71	[22]–M. Naeijian	Whippy Harris Hawks optimization	WHHO	0.76078094	0.228574	1.451895	0.03672887	55.42643282	0.727182	2	0.000774553458233
72		Enriched HHO	EHHO	0.76076901	0.586184	1.968451	0.03659883	55.63943956	0.240965	1.4569104	0.000764087180733
73		Performance-Guided JAYA	PGJAYA	0.7608	0.21031	1.445	0.0368	55.8135	0.88534	2	0.000756809380682
74		Flexible particle swarm optimization	FPSO	0.76078	0.22731	1.4516	0.036737	55.3923	0.72786	1.99969	0.000824115708700
75		Improved JAYA	IJAYA	0.7601	0.00504	1.2186	0.0376	77.8519	0.75094	1.6247	0.000980735473838
**76**		**Bird mating optimizer**	**BMO**	**0.76078**	**0.2111**	**1.44533**	**0.03682**	**55.8081**	**0.87688**	**1.99997**	**0.000754742709027**
77		Generalized oppositional teaching learning based optimization	GOTLBO	0.7608	0.13894	1.7254	0.0365	53.4058	0.26209	1.4658	0.000774762031338
78		Artificial bee swarm optimization	ABSO	0.76078	0.26713	1.46512	0.03657	54.6219	0.38191	1.98152	0.000765494526404
79		Particle swarm optimization algorithm	PSO	0.7623	0.4767	1.5172	0.0325	43.1034	0.0102	2	0.010229431854004
80		Genetic algorithm	GA	0.7608	0.0001	1.3355	0.0364	53.7185	0.0001	1.481	0.359403441383185
81	[23]–D. Saadaoui	Genetic algorithm based on non-uniform nutation	GAMNU	0.760827	0.32245246	1.481028	0.0363644	53.11079	0.00027392	1.470101	0.000795540420037
82		Improved teaching–learning-based optimization	ITBLO	0.7608	0.226	1.451	0.036	55.4854	0.7493	2	0.001243798859174
83		Comprehensive learning PSO	CLPSO	0.7607	0.25843	1.4625	0.0367	57.9422	0.38615	1.9435	0.000788578623039
84		List-based simulated annealing	LBSA	0.7606	0.29814	1.476	0.0363	60.188	0.27096	1.9202	0.000814161645695
85		Teaching learning based optimization	TLBO	0.761	0.2947	1.473	0.0366	53.121	0.1373	1.9938	0.000799018007603
86		Particle swarm optimization with whale optimizer algorithm	PSO-WOA	0.761091	0.20123	1.463321	0.034223	82.82299	0.93611	1.773674	0.001432357624605
87	[24]–Y. Liu	Advanced Slime Mould Algorithm (SMA) integrated Nelder-Mead simplex strategy and Chaotic map	CNMSMA	0.760781	0.225976	1.451017	0.03674	55.48545	0.750681	1.999999	0.000757922728618
88		Improved JAYA	IJAYA	0.760851	0.197606	1.999999	0.036389	56.02268	0.30394	1.476412	0.000794207672981
89		Generalized oppositional teaching learning-based optimization	GOTLBO	0.760565	0.194073	1.44084	0.036599	57.46685	0.635158	1.867391	0.000782760785458
90		Multiple learning backtracking search algorithm	MLBSA	0.760774	0.192658	1.996413	0.036533	54.14372	0.291393	1.472192	0.000769136555275
91		Generalized opposition-based learning mechanism with flower pollination algorithm with Nelder-Mead simplex method	GOFPANM	0.760781	0.225971	0.749382	55.48558	1.451045	0.03674	1.999999	0.627845824154756
92		Slime Mould algorithm	SMA	0.721537	0.749361	1.999999	0.03674	55.48551	0.225972	1.451016	0.035041745078673

**Table 2:** Numerical *RMSE* results in parameters estimation of PWP201 solar module.

Method	Ref./First author	Full title of the algorithm	Short title	*I*_*pv*_ (A)	*I*_01_ (μA)	*n*_1_	*R*_*S*_ (Ω)	*R*_*P*_ (Ω)	*I*_02_ (μA)	*n*_2_	*RMSE*
1	[25]–Premkumar	Improved gradient-based optimization algorithm with chaotic drifts	CGBO	1.0305	3.48	48.6428	1.2013	981.8874	3.89×10^−6^	34.7828	0.002199165255522
2		Gradient-based optimization	GBO	1.0305	3.47	48.6314	1.2016	981.2677	0	50	0.002199691952382
3	[26]–Basset	Equilibrium optimizer	EO	1.0288	9.38×10^−4^	47.1325	1.1896	1310.6705	3.96	49.1369	0.002416169330585
4	[30]–Javidy	Ions motion algorithm	IMO	1.0251	0	45.7618	1.2339	1849.8346	3.07	48.1472	0.003177249323240
5	[15]–M. Rawa	Honey badger algorithm and artificial Gorilla troops optimizer	HBA-GTO	1.03242	2.5130	47.418	1.2393	744.724	3.89×10^−6^	50	0.002076434123783
6		**Artificial Gorilla troops optimizer and Honey badger algorithm**	**GTO-HBA**	**1.0325**	**2.5150**	**47.421**	**1.23928**	**743.666**	**3.88×10** ^**−6**^	**49.88**	**0.002072962280362**

**Table 3:** Numerical *RMSE* results for parameters estimation of MSX–60 module.

Method	Ref./First author	Full title of the algorithm	Short title	*I*_*pv*_ (A)	*I*_01_ (μA)	*n*_1_	*R*_*S*_ (Ω)	*R*_*P*_ (Ω)	*I*_02_ (μA)	*n*_2_	*RMSE*
1	[28]–Kumar	Analytical method	AM	3.8752	3.61×10^−4^	1.0000	0.3084	280.6449	9.38	2.0000	0.025508634055856
2		Numerical method	NM	3.8046	3.99×10^−4^	0.9986	0.3397	280.2171	4.03	2.0014	0.030452568644153
3	[32]–Ishaque	Iteration method	IM	3.8000	4.70×10^−4^	1.0000	0.3500	176.4000	4.70×10^−4^	1.2000	0.077474517740212
4	[31]–Elbaset	Newton method	NewM	3.8084	4.87×10^−4^	1.0003	0.3692	169.0471	6.15×10^−4^	1.9997	0.101470319375997
5	[11]–Ćalasan	Chaotic optimization approach	COA	3.8418	4.96×10^−2^	1.2569	0.2495	267.5700	9.55×10^−9^	1.9345	0.013760760922675
6	[7]–Ćalasan	Success-history-based adaptive differential evolution	CLSHADE	3.812527	0.12311	1.32290	0.226800	800	7.29990×10^−5^	1.98800	0.012028665165887
7	[15]–M. Rawa	Honey badger algorithm and artificial Gorilla troops optimizer	HBA- GTO	3.812527	0.12312	1.32288	0.226805	805.46	7.30×10^−8^	1.98800	0.011955126049429
8		**Artificial Gorilla Troops optimizer and Honey badger algorithm**	**GTO- HBA**	**3.81252689**	**0.1231199**	**1.32286**	**0.226801**	**807.11**	**7.299×10** ^**−5**^	**1.9881**	**0.011896989581563**

**Fig 7:**
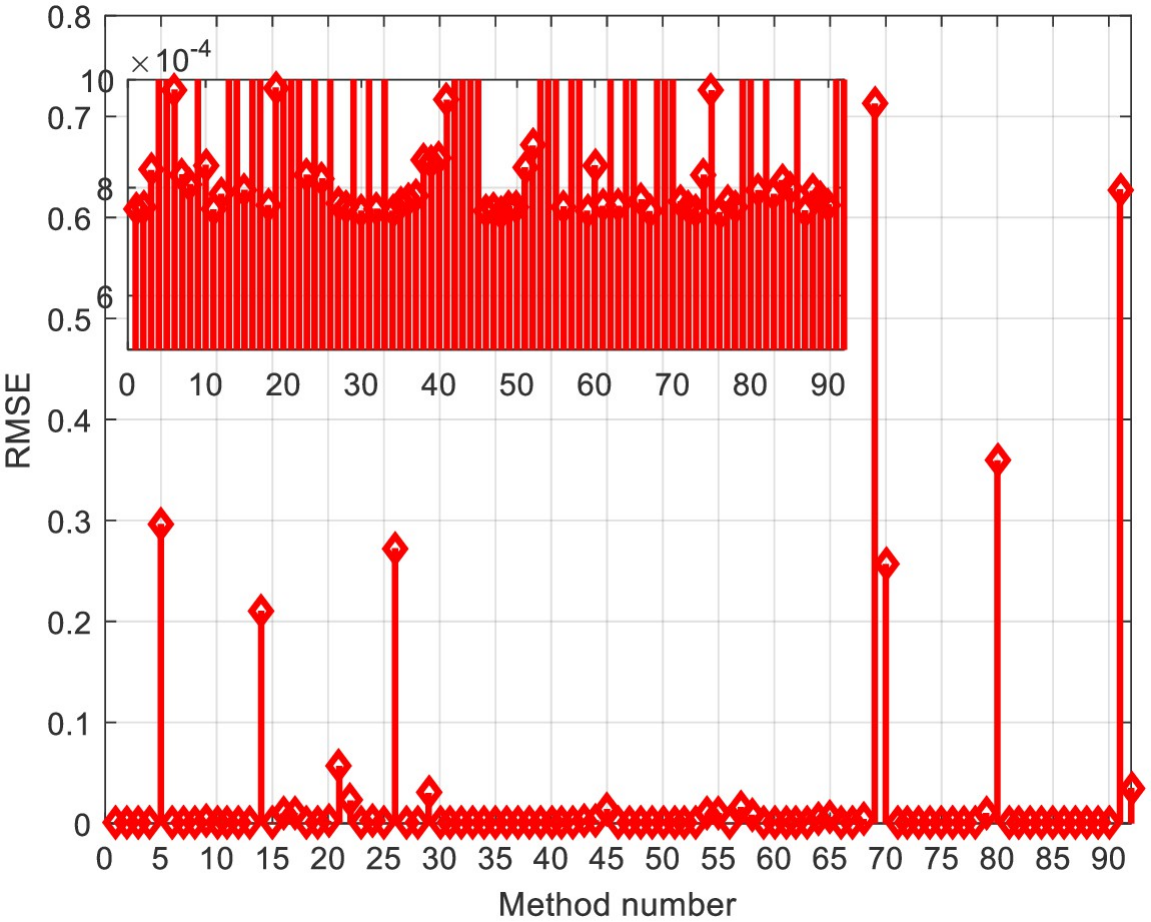
*RMSE* comparison using all investigated methods in Table 1: RTC-F solar cell.

**Fig 8:**
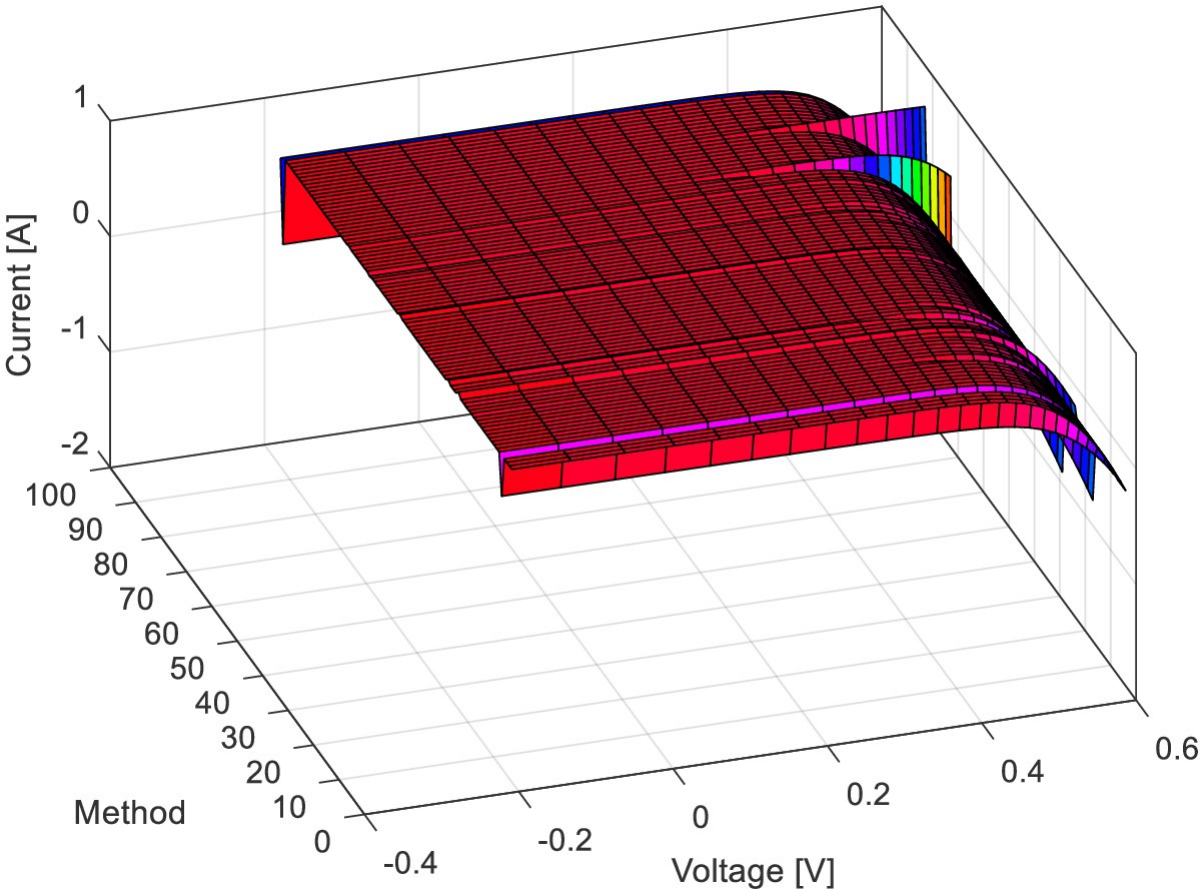
*I*-*U* characteristics using all investigated methods in Table 1: RTC-F solar cell.

For the RTC-F solar cell, 10 publications from the year 2021 were observed, considering all the algorithms that the authors of these papers compared. Fig 7 depicts the *RMSE* values for the RTC-F solar cells across all observed solar cells/modules and various methods/approaches from the literature. The 3D current-voltage-method dependencies of the observed solar cells/modules are shown in Fig 8. The curves for individual cells/modules are approximately identical, confirming the close *RMSE* error values.

Table 1 indicates that the lowest *RMSE* value is 0.0007547427090268051, and the bird mating optimizer (BMO) provides the most accurate parameters. Based on the error and 3D graphs of current-voltage-method characteristics, it is evident that the methods under the ordinal numbers 5, 14, 69, 70, 91, and so on are the least accurate. For the PWP201 module, the minimum error value is 0.002072962280362, obtained by applying the artificial Gorilla Troops optimizer and Honey Badger algorithm (GTO-HBA).

**Fig 9:**
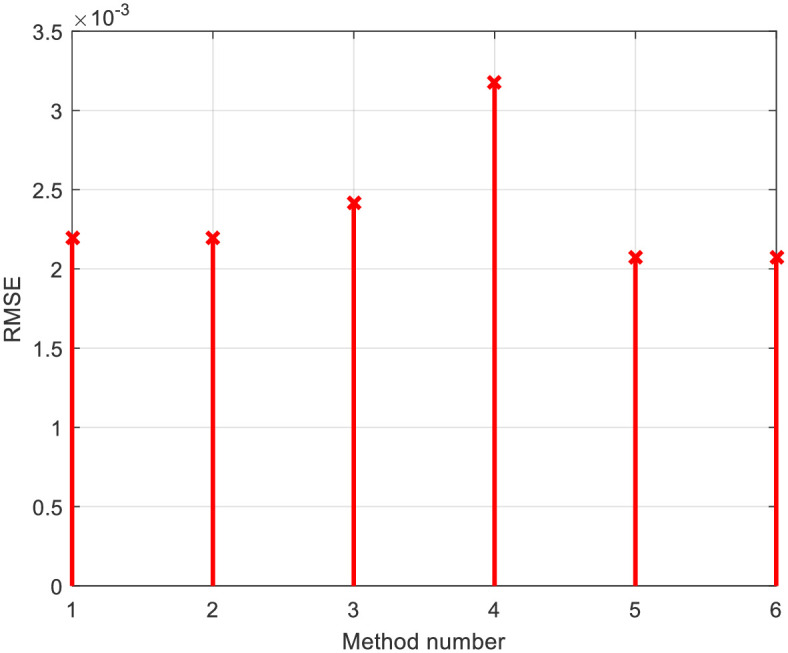
*RMSE* comparison using all investigated methods in Table 2: PWP201 solar module.

**Fig 10:**
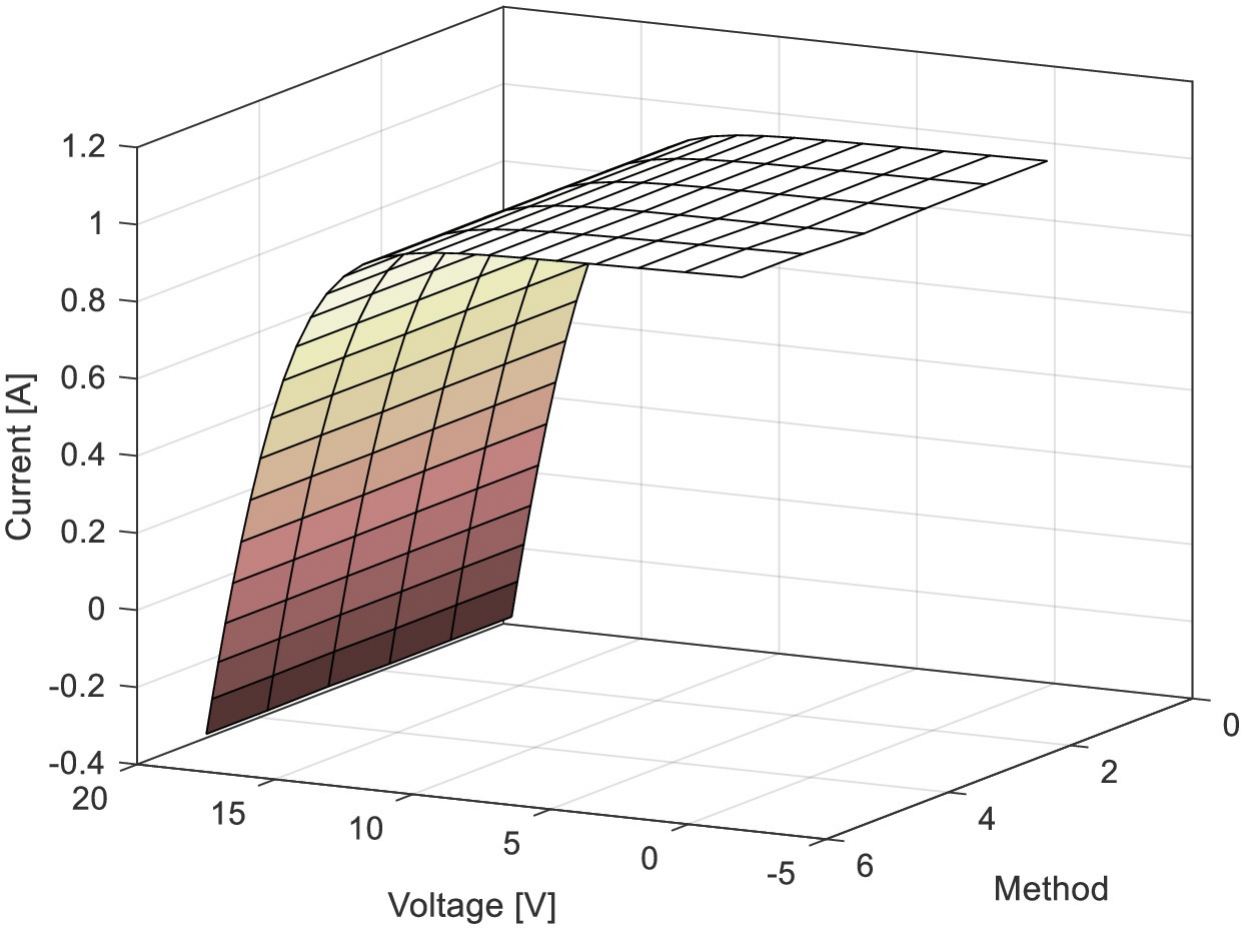
*I*-*U* characteristics using all investigated methods in Table 2: PWP201 solar module.

**Fig 11:**
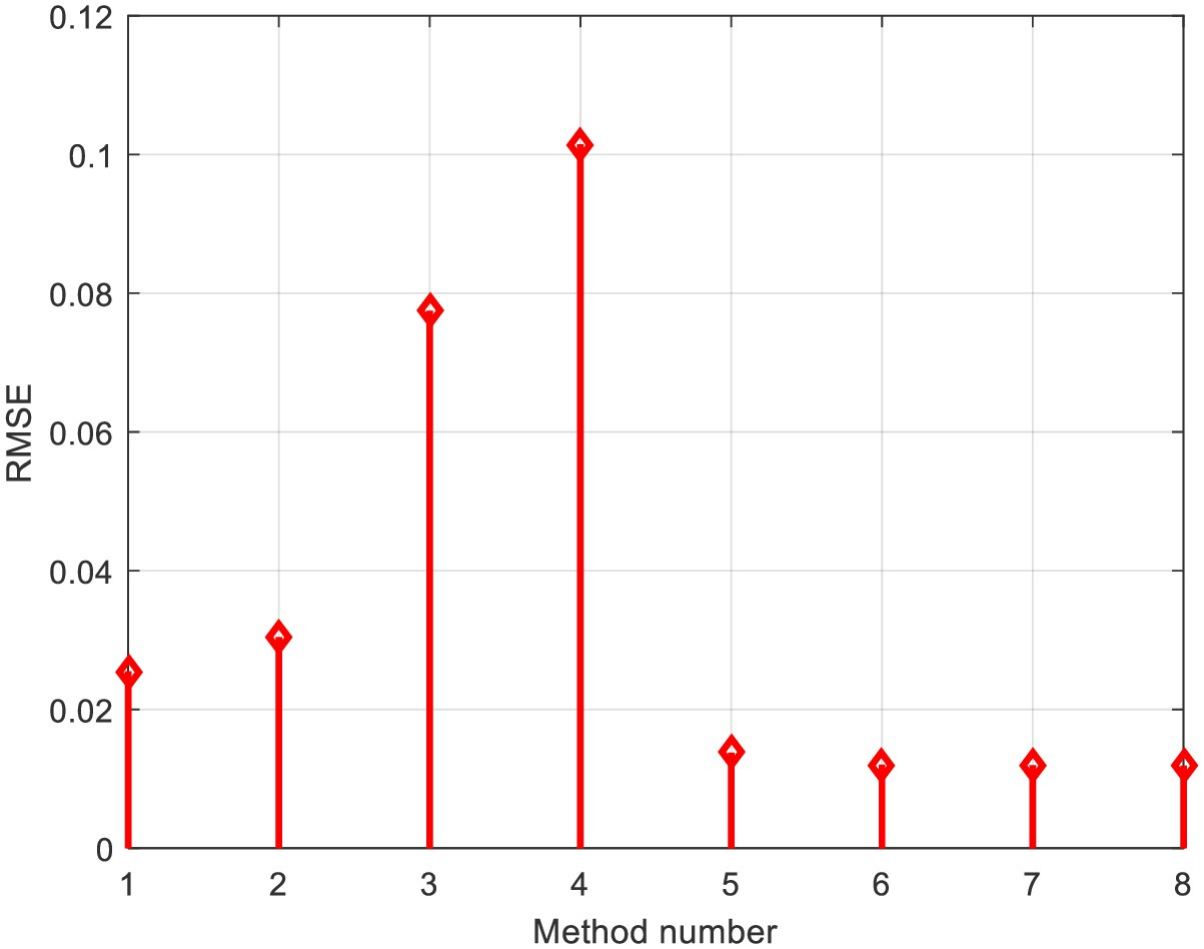
*RMSE* comparison using all investigated methods in Table 3: MSX-60 module.

**Fig 12:**
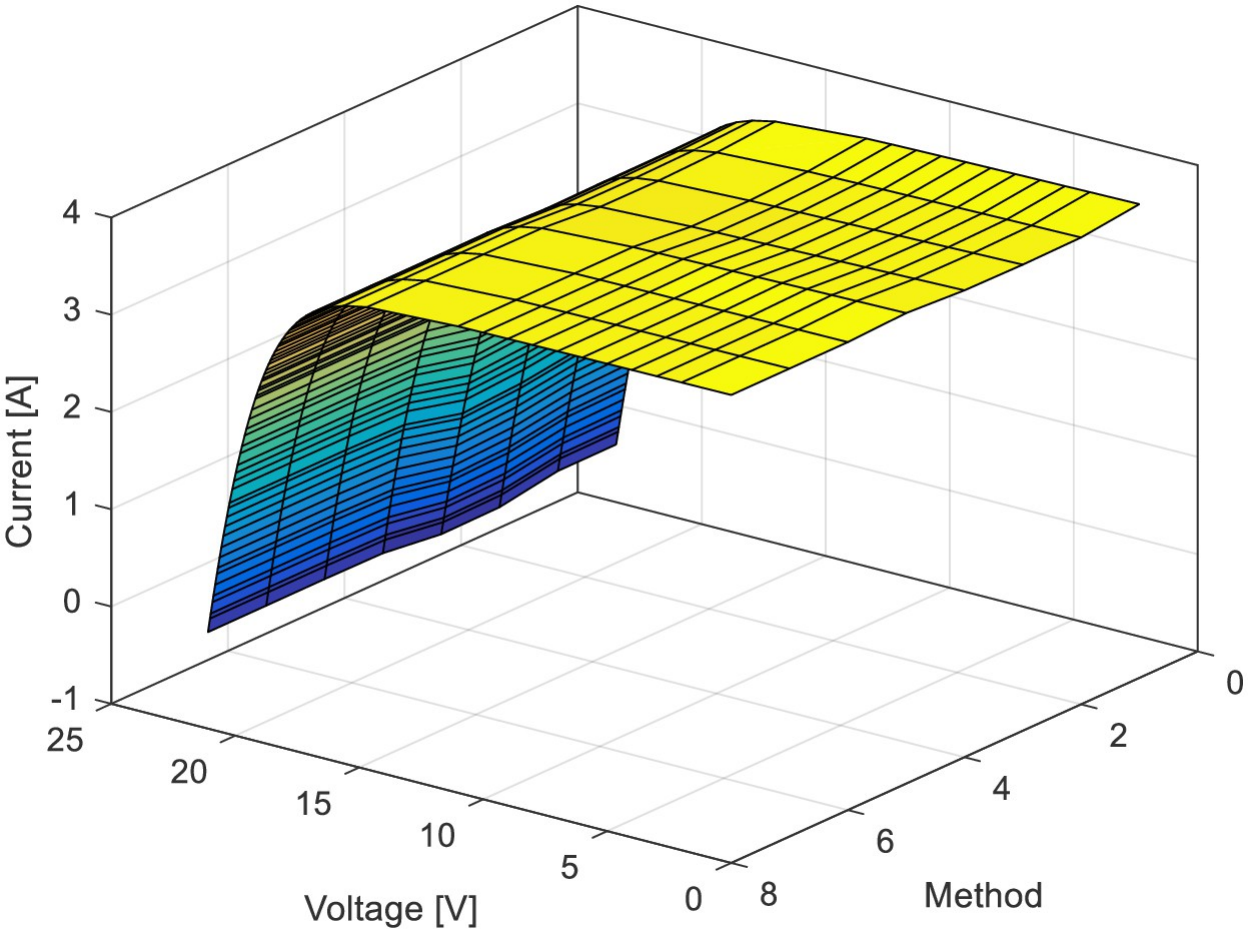
*I*-*U* characteristics using all investigated methods in Table 3: MSX-60 module.

Figs 9 and 10 present the *RMSE* values for all observed methods and the 3D current-voltage-method curves, respectively. Finally, for the MSX-60 PV model, the minimum *RMSE* value is 0.011896989581563, achieved using the same GTO-HBA algorithm. The *RMSE* values for all observed methods and 3D current-voltage-method curves are presented in Figs 11 and 12, respectively.

This section also examines the justification for applying the proposed algorithm and the proposed equivalent circuits of the PV_DDM_ of solar cells. Parameters for both the standard circuit of the double-diode model and the proposed equivalent circuits were estimated, and the obtained results are shown in Table 4.

**Table 4:** Estimated parameters value for the proposed equivalent circuits for all observed solar cells/modules.

Investigated PV cell/module	Model	Parameters							
RTC-F solar cell	Standard PV_DDM_	*I*_*pv*_ (A)	*I*_01_ (μA)	*I*_02_ (μA)	*n*_1_	*n*_2_	*R*_*S*_ (Ω)	*R*_*P*_ (Ω)	*RMSE*
		0.760799044982895	0.16888000000000	0.96456799999999	1.42684474189017	1.95184345106831	0.0371936710473891	55.1802821347085	7.4643×10^−4^
	Proposed DDMR_i_	*I*_*pv*_ (A)	*I*_01_ (μA)	*I*_02_ (μA)	*n*_1_	*n*_2_	*R*_i_ (Ω)	*R*_*P*_ (Ω)	*RMSE*
		0.760267195714892	0.19143721351772	0.80000000000000	1.44115258818800	1.99998087504806	0.0414763685237943	52.1560036764894	7.5296×10^−4^
	Proposed DDMR_D_	*I*_*pv*_ (A)	*I*_01_ (μA)	*I*_02_ (μA)	*n*_1_	*n*_2_	*R*_*D*_ (Ω)	*R*_*P*_ (Ω)	*RMSE*
		0.760265535925480	0.47895034932859	0.63759867004178	1.45000000000000	2	0.0403628702118511	52.4996192896770	7.5447×10^−4^
PWP201 solar module	Standard PV_DDM_	*I*_*pv*_ (A)	*I*_01_ (μA)	*I*_02_ (μA)	*n*_1_	*n*_2_	*R*_*S*_ (Ω)	*R*_*P*_ (Ω)	*RMSE*
		1.03235759399800	2.49659259385554	3.88957851311559×10^−6^	47.3985555810125	47.8155814449944	1.24054729274862	748.323070745754	0.002039992268844
	Proposed DDMR_i_	*I*_*pv*_ (A)	*I*_01_ (μA)	*I*_02_ (μA)	*n*_1_	*n*_2_	*R*_I_ (Ω)	*R*_*P*_ (Ω)	*RMSE*
		1.03064927564657	2.49114224992343	4.80000000000000×10^−6^	47.4753733519336	39.9040000000000	1.24274919592250	749.477455486845	0.002039504798115
	Proposed DDMR_D_	*I*_*pv*_ (A)	*I*_01_ (μA)	*I*_02_ (μA)	*n*_1_	*n*_2_	*R*_*D*_ (Ω)	*R*_*P*_ (Ω)	*RMSE*
		1.03064484103504	6.66702091108575	4.22328916767372×10^−6^	47.4771270094007	59.8560000000000	1.24260427503492	749.563397156701	0.002039991374460
MSX-60 solar module	Standard PV_DDM_	*I*_*pv*_ (A)	*I*_01_ (μA)	*I*_02_ (μA)	*n*_1_	*n*_2_	*R*_*S*_ (Ω)	*R*_*P*_ (Ω)	*RMSE*
		3.81439536827244	0.10425789014917	5.83992800000000×10^−5^	1.31020984316218	1.59040000000000	0.230992245321899	640	0.011583871384354
	Proposed DDMR_i_	*I*_*pv*_ (A)	*I*_01_ (μA)	*I*_02_ (μA)	*n*_1_	*n*_2_	*R*_i_ (Ω)	*R*_*P*_ (Ω)	*RMSE*
		3.81465593921553	0.09848800000000	7.20000000000000×10^−5^	1.30657061249884	1.67999999999505	0.232143472961832	560.990131493344	0.011572680236556
	Proposed DDMR_D_	*I*_*pv*_ (A)	*I*_01_ (μA)	*I*_02_ (μA)	*n*_1_	*n*_2_	*R*_*D*_ (Ω)	*R*_*P*_ (Ω)	*RMSE*
		3.81498301086033	0.19880940872223	1.00040664202577×10^−6^	1.30403890783424	2	0.233003509063835	541.439828616586	0.011571609301025

**Fig 13:**
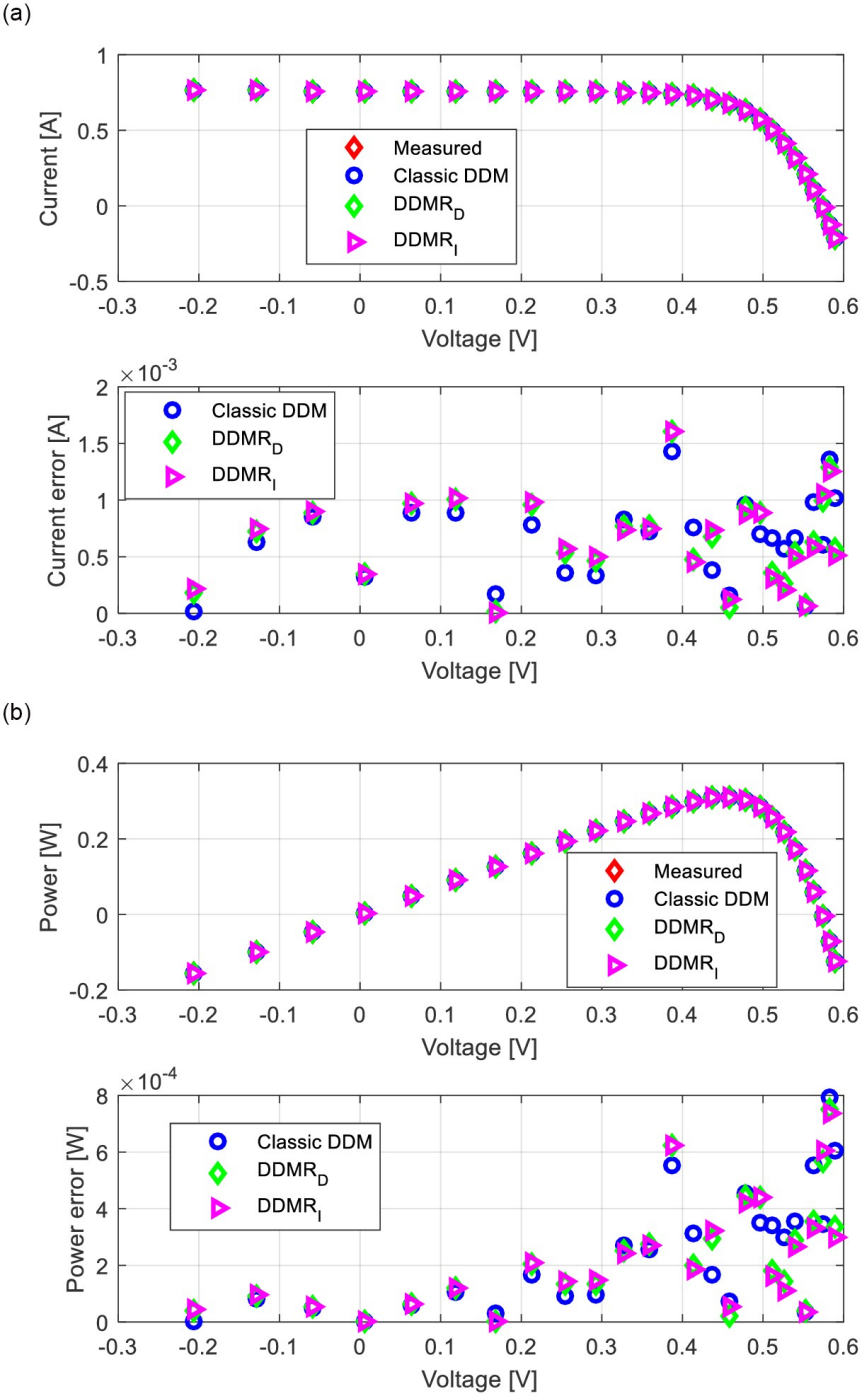
RTC-F solar cell: (a) *I*-*U* and (b) *P*-*U* characteristics.

**Fig 14:**
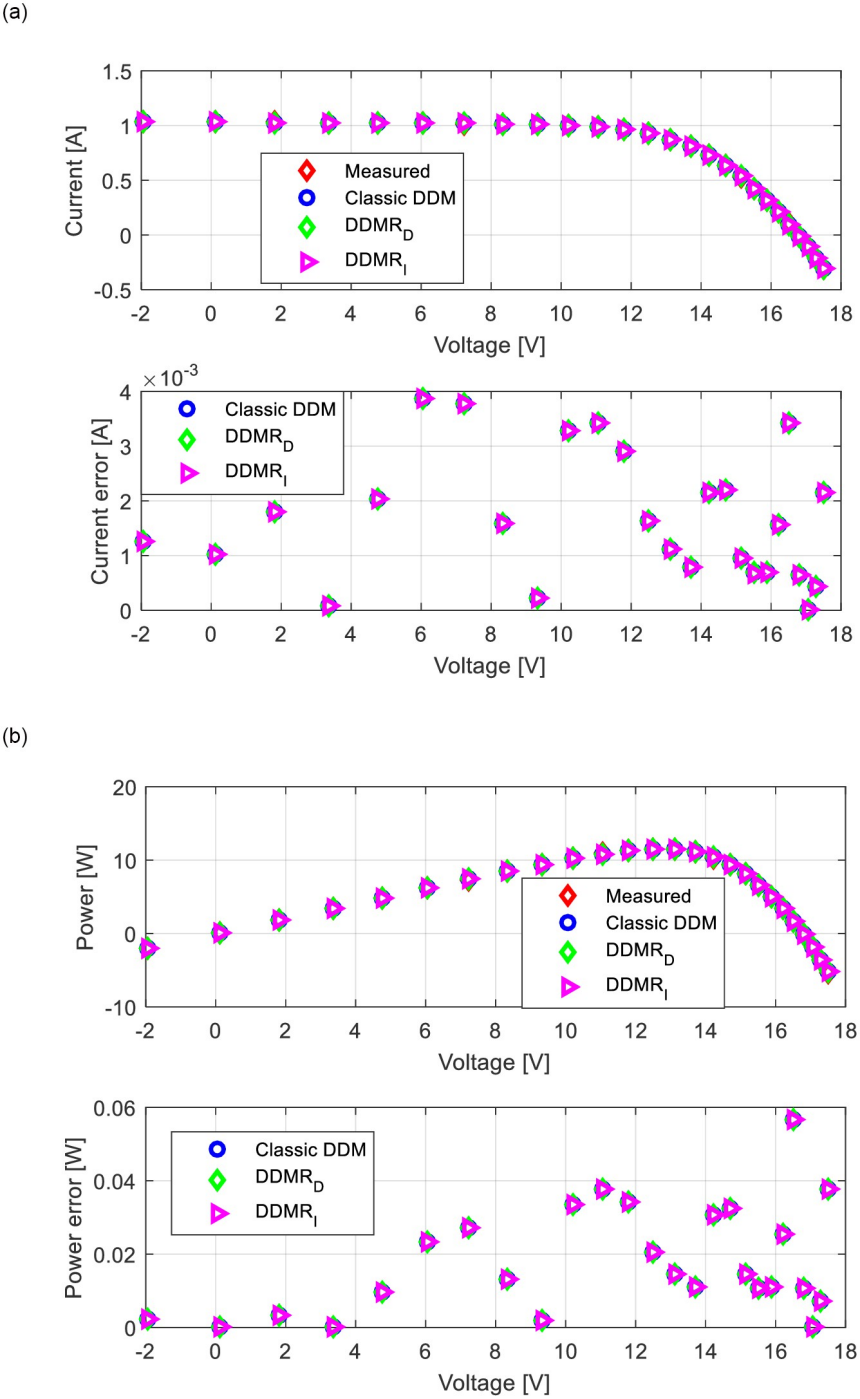
PWP201 solar module: (a) *I*-*U* and (b) *P*-*U* characteristics.

**Fig 15:**
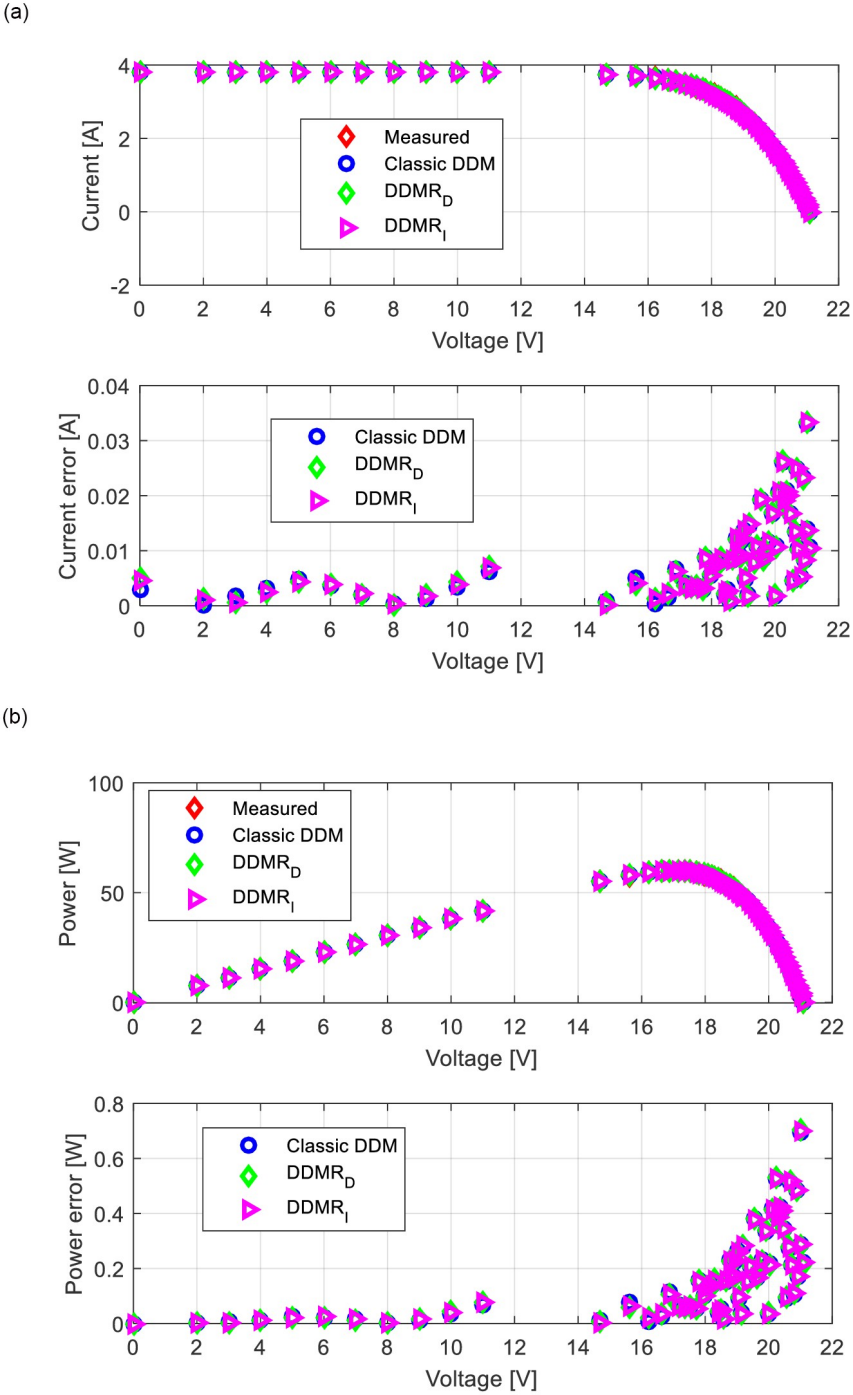
MSX-60 module: (a) *I*-*U* and (b) *P-U* characteristics.

Appropriate *I*-*U* and power-voltage (*P*-*U*) characteristics for all observed solar cells/modules (RTC-F solar cell, PWP201 solar module, and MSX-60 solar module) are shown in Figs 13–15. The corresponding current error voltage and power error voltage characteristics are also presented in the same figures.

Observing the results for standard double-diode models for all solar cells/modules, it is clear that the proposed algorithm is highly efficient in parameter estimation. The application of the proposed algorithm determines solar cell/module parameters that ensure a smaller *RMSE* value than all the approaches presented in the literature.

Regarding the proposed equivalent solar cell circuits, it is evident that they are highly accurate in representing solar cells. Moreover, the accuracy of calculating the parameters of the proposed equivalent solar cell circuits is either identical or even better than the application of standard PV_DDM_ equivalent circuits. In comparison with some literature approaches for the standard PV_DDM_, the proposed equivalent circuit has significantly better accuracy in terms of matching measured and simulated *I*-*U* characteristics (lower *RMSE* value) for all three observed cells/modules. These findings suggest that the standard PV_DDM_ equivalent circuit (shown in Fig 2) does not represent a unique two-diode 7-parameter model of solar cells. However, the proposed PV_DDM_ equivalent circuits, with their analytical closed-form expression for the current as a voltage function, are justified, efficient, and desirable for practical applications.

## 6. Experimental results

The researchers conducted experimental measurements of the *I*-*U* characteristics on a laboratory solar module to further verify the proposed equivalent circuits of the double-diode models of solar cells. For this purpose, an experimental setup was used, consisting of the following components:

- Solar module: Dimensions (W × H × D) 130 × 95 × 65 mm, weight 90 g, power 400 mW, maximum voltage 2.0 V, maximum current 0.5 A
- Insolation definition lamp
- Data acquisition set (USB Data Monitor)
- USB interface
- A PC with embedded software

The USB Data Monitor was used for data acquisition and processing, while the USB interface enabled the connection to the software. The electronic load and the integrated voltage supply could be controlled via the installed software on the PC.

The measurement procedure was as follows:

1. The solar module was irradiated using the lamp (placed at a distance of more than half a meter to prevent overheating), and the insolation value was measured using an insolation meter TES 1333R.
2. The *I*-*U* characteristics for the desired current values were recorded using the installed program (software), as this is precisely what the data acquisition set provides.
3. The measurement was repeated several times, ensuring that the temperature of the solar panels at the beginning and end of the measurement was identical or slightly different (less than 1 degree).
4. The insolation was also changed by adjusting the lamp’s distance. In this case, measurements were performed for three insolation values.

These experimental measurements on the laboratory solar module were conducted to validate further the proposed equivalent circuits of the double-diode models of solar cells.

**Fig 16:**
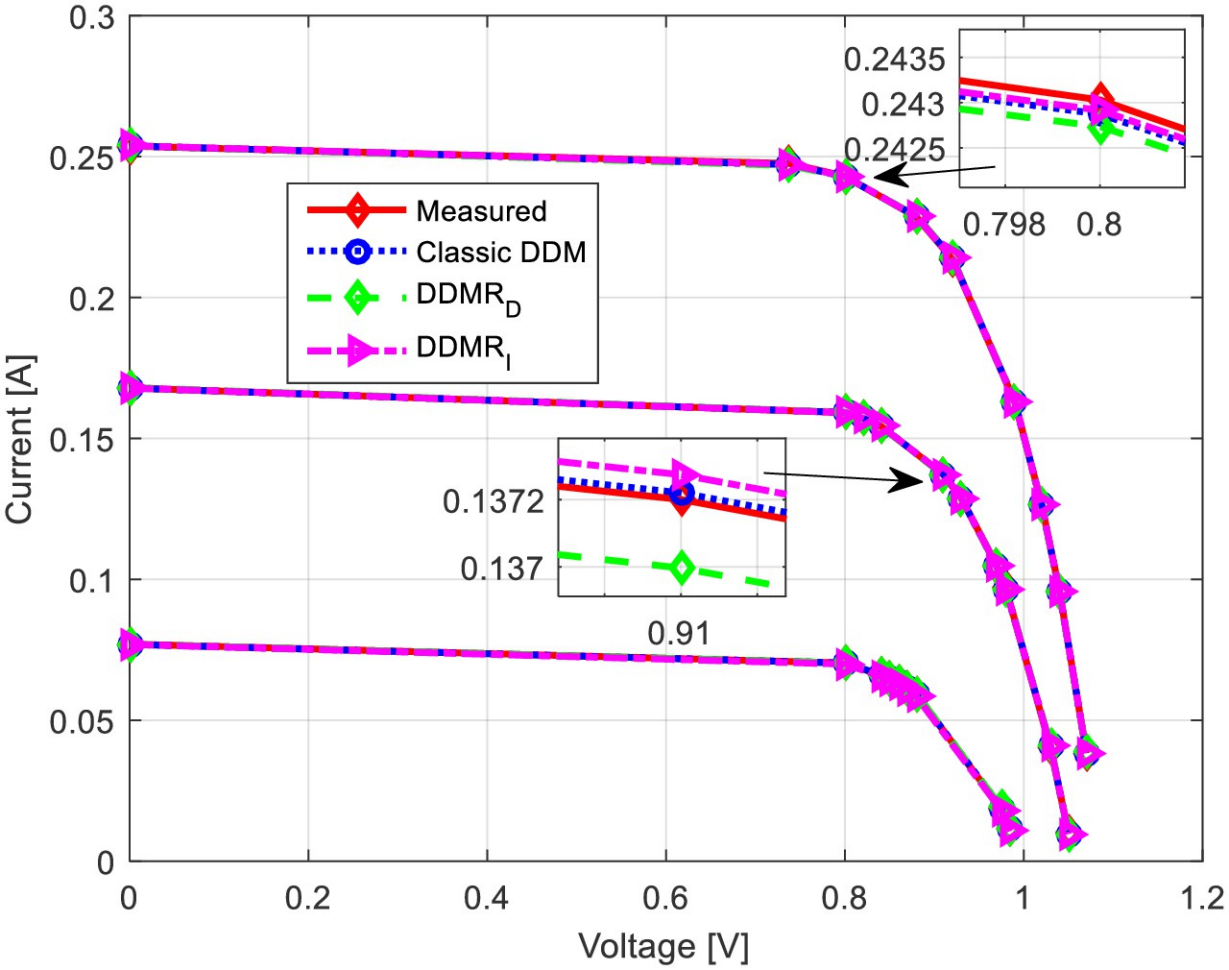
*I*-*U* characteristics of the experimental PV solar module.

**Fig 17:**
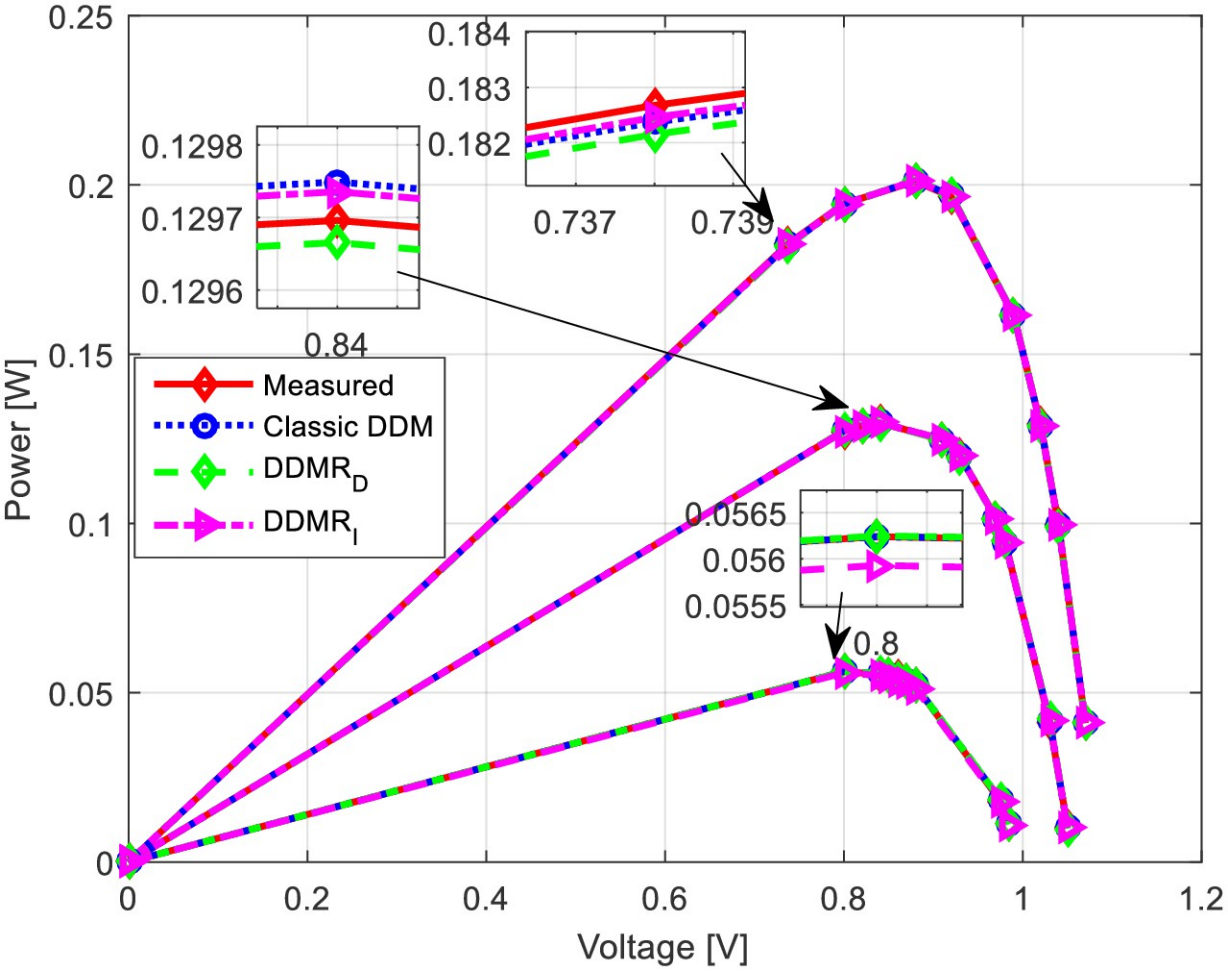
*P*-*U* characteristics of the experimental PV solar module.

Using the measured data, the parameters for both the standard two-diode and the proposed equivalent circuits of the two-diode models of solar cells were estimated for all three insolation values (1130 W/m^2^, 880 W/m^2^, and 610 W/m^2^). The results are shown in Table 5 and graphically in Fig 16 (*I*-*U* characteristics) and Fig 17 (*P*-*U* characteristics).

**Table 5:** Estimated parameters value for proposed equivalent circuits of the experimental solar module.

Irradiance (W/m^2^)	Model	Parameters							
1130	Standard PV_DDM_	*I*_*pv*_ (A)	*I*_01_ (μA)	*I*_02_ (μA)	*n*_1_	*n*_2_	*R*_*S*_ (Ω)	*R*_*P*_ (Ω)	*RMSE*
		0.253965219278999	0.0501892912172816	1.00008495466697×10^−5^	0.327727673466390	0.0710074517700198	0.135101041177656	191.670102441427	2.5639×10^−4^
	Proposed DDMR_i_	*I*_*pv*_ (A)	*I*_01_ (μA)	*I*_02_ (μA)	*n*_1_	*n*_2_	*R*_I_ (Ω)	*R*_*P*_ (Ω)	*RMSE*
		0.253856448878200	0.0611823696360057	1.500000000000005	0.332378057978836	0.272155919032744	0.131632007876652	200.681400657400	1.85×10^−4^
	Proposed DDMR_D_	*I*_*pv*_ (A)	*I*_01_ (μA)	*I*_02_ (μA)	*n*_1_	*n*_2_	*R*_*D*_ (Ω)	*R*_*P*_ (Ω)	*RMSE*
		0.253829282904523	0.0539366966286727	9.31439222759235×10^−6^	0.318434881986277	0.407178337295616	0.146399540040118	165.381719978082	4.15×10^−4^
880	Standard PV_DDM_	*I*_*pv*_ (A)	*I*_01_ (μA)	*I*_02_ (μA)	*n*_1_	*n*_2_	*R*_*S*_ (Ω)	*R*_*P*_ (Ω)	*RMSE*
		0.167896855225257	0.0889644323120276	0.000231573030657567	0.339835183544387	0.580233486719126	0.118727467412515	373.064878550933	2.8849×10^−4^
	Proposed DDMR_i_	*I*_*pv*_ (A)	*I*_01_ (μA)	*I*_02_ (μA)	*n*_1_	*n*_2_	*R*_I_ (Ω)	*R*_*P*_ (Ω)	*RMSE*
		0.167894624782303	0.0704759329593824	0.000500000000000000	0.334647349175342	1.62746460771203	0.127658420558237	321.357001964857	2.7×10^−4^
	Proposed DDMR_D_	*I*_*pv*_ (A)	*I*_01_ (μA)	*I*_02_ (μA)	*n*_1_	*n*_2_	*R*_*D*_ (Ω)	*R*_*P*_ (Ω)	*RMSE*
		0.168076790677137	0.228402452358166	0.000150072627062043	0.358611695782715	1.91630608415845	0.0829301268939921	499.938281371082	3.91×10^−4^
610	Standard PV_DDM_	*I*_*pv*_ (A)	*I*_01_ (μA)	*I*_02_ (μA)	*n*_1_	*n*_2_	*R*_*S*_ (Ω)	*R*_*P*_ (Ω)	*RMSE*
		0.0768930083201141	0.072979409907588	0.000287629867150659	0.335032743696242	1.58646972451315	0.158012537695988	826.695986921679	2.2254×10^−4^
	Proposed DDMR_i_	*I*_*pv*_ (A)	*I*_01_ (μA)	*I*_02_ (μA)	*n*_1_	*n*_2_	*R*_I_ (Ω)	*R*_*P*_ (Ω)	*RMSE*
		0.0768756960128056	0.111983672881024	0.000455550000000000	0.345508001762855	2	0.164450793017236	1000	2.3922×10^−4^
	Proposed DDMR_D_	*I*_*pv*_ (A)	*I*_01_ (μA)	*I*_02_ (μA)	*n*_1_	*n*_2_	*R*_*D*_ (Ω)	*R*_*P*_ (Ω)	*RMSE*
		0.0769939418177960	0.139508889718162	0.000202984542268175	0.348858541391620	1.57072411175853	0.0874202499277467	1000	2.45×10^−4^

Based on the presented results, the proposed models of equivalent circuits of the double-diode model have the same accuracy as the standard PV_DDM_ equivalent circuit. The *I*-*U* and *P*-*U* characteristics obtained using the proposed equivalent circuits closely match those obtained using the standard PV_DDM_ equivalent circuit. Therefore, based on these experimental results, it can be concluded that the proposed equivalent circuits have a justified possibility of application for modeling solar cells with a seven-parameter model. The proposed equivalent circuits provide a comparable level of accuracy to the standard PV_DDM_ equivalent circuit in representing the behavior of the solar cells/modules under different insolation conditions.

## 7. Algorithm testing

This section compares the proposed algorithm’s performance against three well-known algorithms in the literature: the standard HBA, the classical particle swarm optimization (PSO), and the evaporation rate-based water cycle algorithm (ERWCA). All results were tested on the same computer and MATLAB environment under the same operating conditions. Based on the results presented in Table 6, the proposed algorithm clearly outperforms the other three algorithms, followed by the ERWCA.

**Table 6:** Algorithm testing.

Algorithm	Best	Worst	Mean	Median	Standard deviation
CHBA	7.4994×10^−4^	0.0010	8.3443×10^−4^	7.6056×10^−4^	1.1334×10^−4^
HBA	7.5674×10^−4^	0.0020	0.0011	8.4289×10^−4^	4.7679×10^−4^
PSO	8.0438×10^−4^	0.0020	0.0011	9.8242×10^−4^	3.7356×10^−4^
ERWCA	7.5044×10^−4^	0.0010	8.1441×10^−4^	7.6249×10^−4^	1.0743×10^−4^

To further evaluate the statistical significance of the differences, the researchers conducted Wilcoxon’s test, a non-parametric statistical test used to compare the performance of different methods. The results of the Wilcoxon’s test are shown in Table 7. According to Wilcoxon’s test results, a value of 0 indicates a significant difference between the methods, while a value closer to 1 suggests a smaller difference. The results in Table 7 show that most of the data ranges between 0 and 1, which means that the proposed method is effective and there is a statistically significant difference between it and the other methods.

**Table 7:** Wilcoxon test comparisons.

*p*-value	CHBA vs. HBA	CHBA vs. PSO	CHBA vs ERWCA
	0.1405	0.0173	0.9097

**Fig 18:**
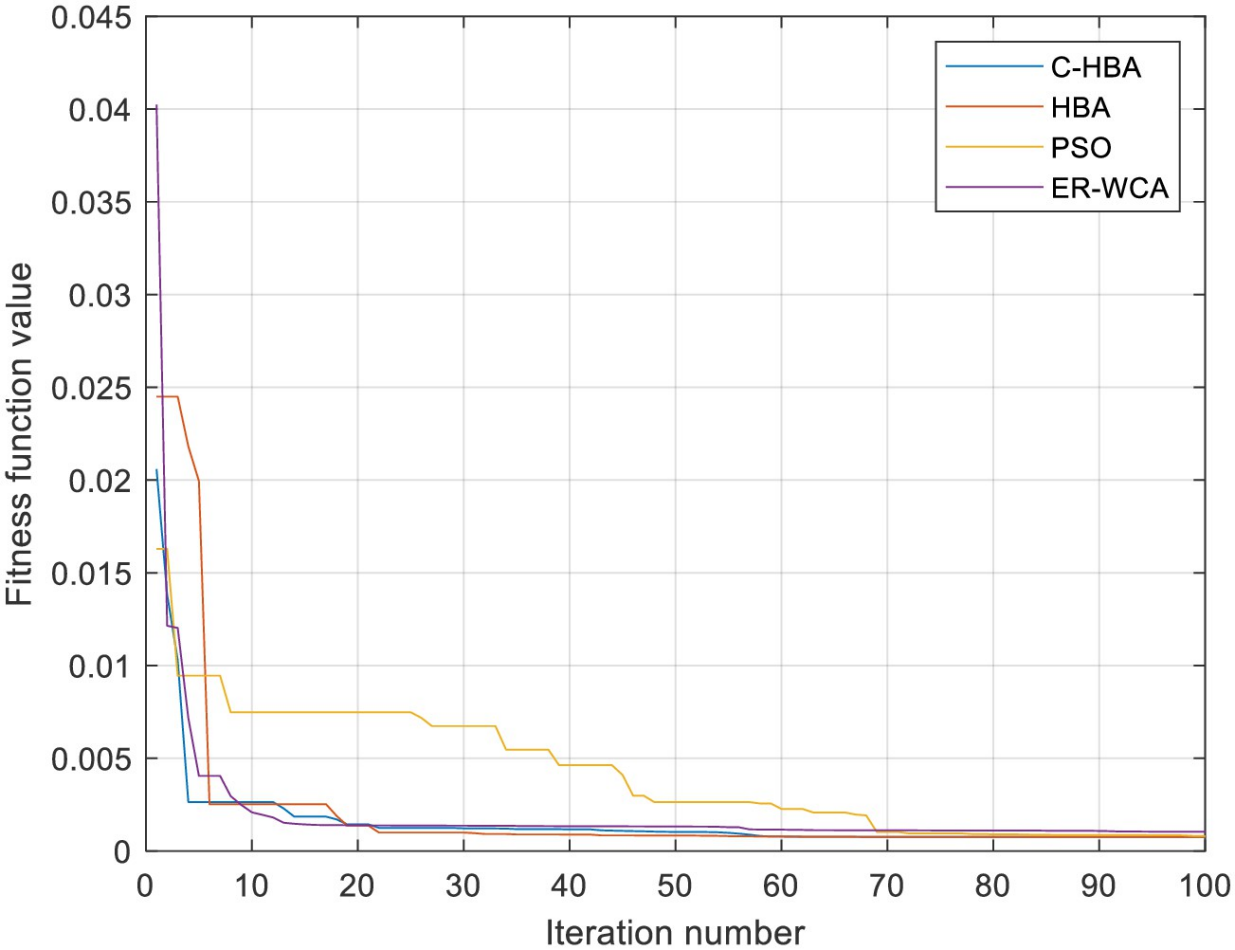
Convergence curves of the investigated algorithms.

Finally, Fig 18 presents the algorithms’ convergence characteristics. This figure clearly demonstrates the proposed algorithm’s superior convergence performance compared to the other three methods. The proposed algorithm exhibits faster convergence and better final solution quality, highlighting its effectiveness in the given application. The experimental results and statistical analysis confirm that the proposed algorithm outperforms the standard HBA, classical PSO, and ERWCA algorithms in terms of solution quality and convergence characteristics.

## 8. Conclusions

This paper addresses the prevalent research direction of modeling solar cells and estimating their parameters. The core novelties of this work are summarized as follows:

- Proposal of two new equivalent circuits of the double-diode solar cell model: Unlike the standard equivalent circuit, the proposed equivalent circuits have an analytical dependence on current and voltage in closed form.
- Derivation and presentation of mathematical relations via the STFT approach for both proposed equivalent circuits.
- Proposal of a chaotic metaheuristic algorithm for estimating solar cell parameters.

The proposed algorithm’s accuracy, applicability, justification, and equivalent schemes were tested on three standard solar cells/modules and measured data from an actual solar module. The key findings are:

- The matching between measured and simulated *I*-*U* curves obtained for the proposed equivalent circuits is identical to or better than the application of the standard equivalent circuit of the double-diode solar cell model.
- This suggests that the well-known equivalent circuit of the double-diode model of solar cells is not a unique seven-parameter model with two diodes and two resistors.
- The proposed equivalent circuits demonstrate the possibility of applying alternative models with the same or better accuracy compared to the standard double-diode model.

In future works, the authors plan to focus on developing new three-diode and *n*-diode models of solar cells, building upon the insights gained from this study. Lastly, this work contributes novel equivalent circuit topologies and a new parameter estimation algorithm, which expands the options available for accurately modeling and analyzing solar cell characteristics.

## Supporting information

S1 File Main parts of the MATLAB code. Voltage-currents characteristics.
(PDF)
