# Supplementary material for: Analytical modeling of novel equivalent circuits of double diode solar cell circuits using a special transcendental function approach
Source: PLoS One. 2024 Nov 14;19(11):e0313713. doi: 10.1371/journal.pone.0313713 (PMC11563384; doi:10.1371/journal.pone.0313713)
Supplement: S1 File — Voltage-currents characteristics. (PDF) [file pone.0313713.s001.pdf]

S1 File. Main parts of the MATLAB code. Voltage-currents characteristics.

Main parts of the MATLAB code for presenting voltage-currents characteristics for both models.

```
% MODEL WITH RD
% define solar cell parameters
% saturation current - Io1, Io2, ideality factor - a1,a2, resistance R, parallel resistance Rp,
% thermal voltage - Vt, photocurrent - Ipv
% load array of measured data for voltage (Umeas) and current (Imeas)
for t=1:length(Imeas)
    beta1=R*Io1/a1/Vt*exp((Umeas(t)+R*Io1)/a1/Vt);
    teta1=lambertw(beta1);
    currentsimulatedRD(t)=Ipv+Io1-Io2*(exp(Umeas(t)/a2/Vt)-1)-Umeas(t)/Rp-a1*Vt/R*teta1;
    errorRD(t)= (Imeas(t)-currentsimulatedRD(t))^2;
 end
RMSE_RD=sqrt(sum(errorRD)/length(Umeas)) ;
% MODEL WITH RI
% define solar cell parameters
% saturation current - Io1, Io2, ideality factor - a1,a2, resistance R, parallel resistance Rp,
% thermal voltage - Vt, photocurrent - Ipv
% load array of measured data for voltage (Umeas) and current (Imeas)
for t=1:length(Imeas)
    beta1=R*Io1/a1/Vt*exp((Umeas(t)+R*(Io1+Ipv))/a1/Vt);
    teta1=lambertw(beta1);
    currentsimulatedRI(t)=Ipv+Io1-Io2*(exp(Umeas(t)/a2/Vt)-1)-Umeas(t)/Rp-a1*Vt/R*teta1;
    errorRI(t)= (Imeas(t)-currentsimulatedRI(t))^2;
end
RMSE_RD=sqrt(sum(errorRI)/length(Umeas));
```
